# Long-term safety and efficacy of cipaglucosidase alfa plus miglustat in individuals living with Pompe disease: an open-label phase I/II study (ATB200-02)

**DOI:** 10.1007/s00415-023-12096-0

**Published:** 2023-12-06

**Authors:** Barry J. Byrne, Benedikt Schoser, Priya S. Kishnani, Drago Bratkovic, Paula R. Clemens, Ozlem Goker-Alpan, Xue Ming, Mark Roberts, Matthias Vorgerd, Kumaraswamy Sivakumar, Ans T. van der Ploeg, Mitchell Goldman, Jacquelyn Wright, Fred Holdbrook, Vipul Jain, Elfrida R. Benjamin, Franklin Johnson, Sheela Sitaraman Das, Yasmine Wasfi, Tahseen Mozaffar

**Affiliations:** 1 University of Florida, Gainesville, FL USA; 2 Friedrich-Baur-Institute at the Department of Neurology, LMU University Hospital, LMU Munich, Munich, Germany; 3 Duke University Medical Center, Durham, NC USA; 4 PARC Research Clinic, Royal Adelaide Hospital, Adelaide, SA Australia; 5 Department of Neurology, University of Pittsburgh School of Medicine and VA Pittsburgh Healthcare System, Pittsburgh, PA USA; 6 Lysosomal and Rare Disorders Research and Treatment Center, Fairfax, VA USA; 7 Neurology, Rutgers New Jersey Medical School, Newark, NJ USA; 8 Guam Regional Medical City, Dededo, Guam; 9 Salford Royal NHS Foundation Trust, Salford, UK; 10 Department of Neurology, University Hospital Bergmannsheil, Heimer Institute for Muscle Research, Bochum, Germany; 11 Neuromuscular Clinic and Research Center, Phoenix, AZ USA; 12 Erasmus MC University Medical Center, Rotterdam, Netherlands; 13 Amicus Therapeutics, Inc., Princeton, NJ USA; 14 University of California, Irvine, Irvine, CA USA

**Keywords:** Glycogen storage disease type II, Alpha glucosidases, Myozyme, *n*-Butyldeoxynojirimycin, Lysosomal storage diseases, Pharmacokinetics

## Abstract

Cipaglucosidase alfa plus miglustat (cipa + mig) is a novel, two-component therapy for Pompe disease. We report data from the Phase I/II ATB200-02 study for up to 48 months of treatment. Four adult cohorts, including one non-ambulatory ERT-experienced (*n* = 6) and three ambulatory cohorts, (two enzyme replacement therapy [ERT]-experienced cohorts [2–6 years (*n* = 11) and ≥ 7 years (*n* = 6)]), one ERT-naïve cohort (*n* = 6), received 20 mg/kg intravenous-infused cipa plus 260 mg oral mig biweekly. Change from baseline (CFBL) for multiple efficacy endpoints at 12, 24, 36, and 48 months, pharmacodynamics, pharmacokinetics, safety, and immunogenicity data were assessed. Six-minute walking distance (% predicted) improved at 12, 24, 36, and 48 months: pooled ambulatory ERT-experienced cohorts, mean(± standard deviation [SD]) CFBL: 6.1(± 7.84), *n* = 16; 5.4(± 10.56), *n* = 13; 3.4(± 14.66), *n* = 12; 5.9(± 17.36), *n* = 9, respectively; ERT-naïve cohort: 10.7(± 3.93), *n* = 6; 11.0(± 5.06), *n* = 6; 9.0(± 7.98), *n* = 5; 11.7(± 7.69), *n* = 4, respectively. Percent predicted forced vital capacity was generally stable in ERT-experienced cohorts, mean(± SD) CFBL − 1.2(± 5.95), *n* = 16; 1.0(± 7.96), *n* = 13; − 0.3(± 6.68), *n* = 10; 1.0(± 6.42), *n* = 6, respectively, and improved in the ERT-naïve cohort: 3.2(± 8.42), *n* = 6; 4.7(± 5.09), *n* = 6; 6.2(± 3.35), *n* = 5; 8.3(± 4.50), *n* = 4, respectively. Over 48 months, CK and Hex4 biomarkers improved in ambulatory cohorts. Overall, cipa + mig was well tolerated with a safety profile like alglucosidase alfa. ATB200-02 results show the potential benefits of cipa + mig as a long-term treatment option for Pompe disease. *Trial registration number*: NCT02675465 January 26, 2016.

**Supplementary Information:**

The online version contains supplementary material available at 10.1007/s00415-023-12096-0.

## Introduction

Pompe disease is a rare, multisystemic, and progressive lysosomal disorder caused by inherited pathogenic variants in the acid α-glucosidase (*GAA*) gene [1, 2]. This results in partial-to-full deficiency of the GAA enzyme that is responsible for the breakdown of lysosomal glycogen [1]. The functional deficiency of GAA leads to the accumulation of lysosomal glycogen, dysregulated autophagy and cellular damage in many tissues, most prominently skeletal muscle, smooth muscle and, in infantile-onset Pompe disease (IOPD), cardiac muscle [1]. Pompe disease can be broadly classified into two subtypes: IOPD and late-onset Pompe disease (LOPD) [3, 4]. The latter is generally less severe and slower progressing, with a broad phenotypic spectrum and residual GAA enzyme activity reported to be between ~ 1% and 30–40% [3–7]. Nonetheless, LOPD remains a debilitating disorder that eventually leads to wheelchair use and ventilator-assisted breathing and places a significant burden on patients, families, and healthcare systems [5, 6, 8].

For many patients with LOPD, enzyme replacement therapy (ERT) initially improves parameters such as muscle strength, motor skills, and pulmonary function [9–12]. Alglucosidase alfa, a recombinant human GAA (rhGAA), was the first approved Pompe disease–specific ERT [3, 13–17]. However, treatment with alglucosidase alfa does not prevent disease progression [9]. A plateau is typically reached 2–3 years after treatment initiation, followed by progressive decline [9–12, 18, 19], highlighting a critical unmet need for new therapies with a more durable treatment response. The suboptimal outcomes observed with alglucosidase alfa led to research to better understand the possible mechanistic challenges of delivering a rhGAA to skeletal muscle [20, 21]. First, rhGAA, which is most active at lysosomal pH, is less stable at the near-neutral pH of the bloodstream, resulting in rapid inactivation after infusion [20]. Second, less than 1% of rhGAA reaches skeletal muscle, meaning that efficient uptake requires that the rhGAA contains bis-phosphorylated mannose-6-phosphate (bis-M6P) *N*-glycans for high-affinity uptake through the cation-independent M6P receptor (CI-MPR) [20]. Lastly, an rhGAA requires processing (proteolytic and *N*-glycan trimming) to enhance enzyme activity and affinity for the glycogen substrate [21]. The need to improve cellular enzyme uptake and glycogen clearance in target tissue to enhance clinical outcomes has led to the development of next-generation ERTs for Pompe disease [20, 22–27].

Cipaglucosidase alfa plus miglustat (cipa + mig) is a novel, two-component Pompe disease therapy designed to address these challenges and ensure efficient rhGAA delivery to the muscle [28, 29]. Cipaglucosidase alfa is enriched with Chinese hamster ovary (CHO) cell (naturally)-derived bis-M6P *N*-glycans to mediate high-affinity binding and effective uptake into muscle via CI-MPR. [30, 31]. Inside the cell, enzyme activity is maximized due to enhanced CHO cell-derived glycosylation; this ensures both proteolytic and *N*-glycan processing into a mature form of the enzyme that has maximal catalytic activity [21, 28]. In addition, the amount of rhGAA available for uptake and processing is maximized by adding the enzyme stabilizer, miglustat [28, 29, 31]. This stabilizes cipaglucosidase alfa in the circulation so that catalytic activity is maintained upon uptake into muscles—where miglustat detaches from cipaglucosidase alfa in muscle cell lysosomes—as well as increasing biodistribution of the enzyme [28, 31].

The Phase III PROPEL clinical study compared the efficacy and safety of cipa + mig and alglucosidase alfa in adults with LOPD [32]. Overall, patients treated with cipa + mig (*n* = 85) showed greater clinically meaningful improvements in absolute and % predicted 6-min walk distance (6MWD) and % predicted forced vital capacity (FVC); however, differences in absolute and % predicted 6MWD did not reach statistical significance, and the difference in % predicted FVC was nominally statistically significant. Nominal significance was also reached in the ERT-experienced group for both 6MWD and FVC [32].

Here we present data from the ongoing, open-label, phase I/II ATB200-02 clinical trial that aims to evaluate the long-term (up to 48 months) efficacy of cipa + mig in adults with Pompe disease. Pharmacodynamics (PD), pharmacokinetics (PK), safety, and immunogenicity were also evaluated.

## Methods

### Study design

**Fig. 1:**
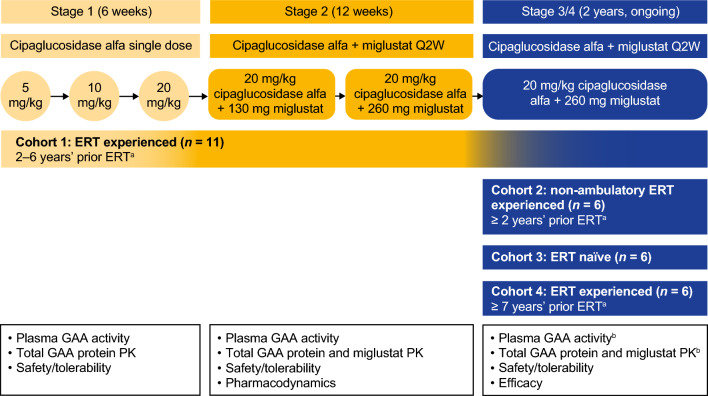
ATB200-02 study design: 18-week primary treatment period with long-term extension. Stage 3 was a 2-year treatment period during which patients received 20 mg/kg of IV-infused cipaglucosidase alfa co-administered with 260 mg of miglustat administered orally every 2 weeks. Stage 4 is an ongoing long-term extension to provide additional safety and efficacy data. ^a^With 20 mg/kg alglucosidase alfa Q2W; ^b^PK assessments for GAA activity; total GAA protein and miglustat in stage 3 were only performed for the first and third doses for cohort 3. Sparse blood sampling for total GAA protein was performed after ≥ 18 months of treatment in cohorts 1 and 3. If a patient had completed stage 3, sparse PK sampling was performed in stage 4. *ERT* enzyme replacement therapy, *GAA* acid α-glucosidase, *IV* intravenous, *PK* pharmacokinetics, *Q2W* every 2 weeks

The ATB200-02 (NCT02675465) study is being conducted at 17 sites in six countries (Australia, Germany, the Netherlands, New Zealand, the UK, and the US). The first patient was enrolled in April 2016. The study design is depicted in Fig. 1. The primary treatment period consisted of stage 1 (6 weeks) and stage 2 (12 weeks). Stage 3 was a 2-year treatment period and stage 4 is an ongoing long-term extension (Fig. 1). The primary objective was to evaluate the safety and tolerability of cipa + mig; the primary objectives of stages 3 and 4 included the evaluation of long-term efficacy.

### Study participants

The study enrolled four cohorts of patients 18–75 years old (18–65 years in cohorts 1–3; 18–75 years in cohort 4) with a diagnosis of Pompe disease based on documented deficiency of GAA enzyme activity or by two pathogenic variants in the *GAA* gene:

- *Cohort 1: ERT experienced (2–6 years), ambulatory* (2–6 years prior ERT with alglucosidase alfa every 2 weeks; 6MWD: 200–500 m; upright FVC: 30–80% of predicted normal value)
- *Cohort 2: ERT experienced (≥ 2 years), non-ambulatory* (≥ 2 years prior ERT with alglucosidase alfa; wheelchair use and unable to walk unassisted)
- *Cohort 3: ERT naïve, ambulatory* (6MWD: 200–500 m; upright FVC: 30–80% of predicted normal value)
- *Cohort 4: ERT experienced (≥ 7 years), ambulatory* (≥ 7 years prior ERT with alglucosidase alfa every 2 weeks; 6MWD: 75–600 m; upright FVC: 30–85% of predicted normal value)

Key exclusion criteria included prior use of any investigational therapy, including adjunctive therapy for Pompe disease within 30 days or five half-lives of the therapy or treatment, whichever was longer; requirement for invasive ventilatory support or non-invasive ventilatory support ≥ 6 h a day while awake (except cohort 2); history of anaphylactic reaction to alglucosidase alfa (except cohort 3); history of high sustained anti-rhGAA antibodies (except cohort 3); presence of active systemic autoimmune disease (all patients with autoimmune disease must have been discussed with the sponsor’s medical monitor). Full inclusion and exclusion criteria are provided in Supplementary Table S1.

### Treatments

Across all study stages, cipaglucosidase alfa was intravenously (IV)-infused every 2 weeks over approximately 4 h (± 15 min) with gradually increased infusion rates; the patient’s body weight determined the total volume of infusion. The infusion was performed at a hospital or infusion center by qualified study personnel; for some eligible patients, the infusion could be performed at home by a trained home-infusion nurse. Changes in the duration of cipaglucosidase alfa infusion due to safety or tolerability issues were documented. In stages 2, 3, and 4, miglustat oral capsules were administered 1 h prior to infusion of cipaglucosidase alfa, with a 2-h fasting period before and after miglustat administration.

The dosing regimen was determined by evaluating single and multiple ascending doses in the initial cohort of ERT-experienced ambulatory patients (cohort 1) using a sentinel dosing approach at each study stage (Fig. 1). In stage 1, cohort 1 received cipaglucosidase alfa as a single agent (5, 10, or 20 mg/kg). In stage 2, cohort 1 received cipaglucosidase alfa 20 mg/kg co-administered with miglustat 130 mg or 260 mg for three consecutive doses each. Once an appropriate regimen had been determined, and safety assessed by the external data monitoring committee, the additional cohorts of non-ambulatory (cohort 2), ERT-naïve (cohort 3), and ERT-experienced (cohort 4) patients were enrolled into stage 3. In stages 3 and 4, all cohorts received cipaglucosidase alfa 20 mg/kg co-administered with miglustat 260 mg. A sentinel dosing approach was used for cohorts 2 and 3 but not cohort 4, as this cohort had similar baseline characteristics to cohort 1 and was enrolled at a later stage when almost 2 years of safety data for cohort 1 were available.

### Assessments and outcomes

Efficacy outcomes, assessed over 48 months, included the change from baseline (CFBL) in 6MWD (% predicted and absolute CFBL in meters), % predicted sitting FVC, manual muscle test (MMT), patient-reported outcomes (PROs; Rasch-built Pompe-specific Activity [R-PAct] scale, Rotterdam Handicap Scale [RHS] and Fatigue Severity Scale [FSS]) and PD outcomes (the biomarkers urine glucose tetrasaccharide [Hex4] and serum creatine kinase [CK]). Efficacy was assessed every 3 months in stage 3, and every 6 months in stage 4. In addition, Subject and Physician Global Impression of Change (SGIC and PGIC, respectively) were completed during stages 3 and 4. Safety evaluations included adverse event (AE) monitoring (including infusion-associated reactions [IARs]), clinical laboratory profiles (serum chemistry, hematology, and urinalysis), vital signs, 12-lead electrocardiograms, and physical examinations. Further details on efficacy and safety assessments are provided in Supplementary Sect. S1. In addition, blood and urine sampling was carried out to enable characterization of PD (urinary Hex4 and serum CK) measurements, PK (plasma total GAA protein and plasma miglustat) parameters, and immunogenicity (anti-cipaglucosidase alfa [drug] antibodies [ADAs], neutralizing antibodies [NAbs], antibodies cross-reactive to alglucosidase alfa, and anti-rhGAA immunoglobulin E [IgE] antibodies) measurements (Supplementary Section S1). PK data from stage 1, cohort 1 are reported here and full PK details are provided in Supplementary Section S2.

The COVID-19 pandemic had minimal impact on the study with respect to missed infusions and appointments.

### Data analysis

No formal sample size calculation was performed because this study began as a first-in-human trial, which did not involve statistical testing of any hypothesis or comparison. However, a sample size of between 18 and 34 patients was considered adequate for this study. No inferential statistics were used in this study. In general, continuous variables were summarized using descriptive statistics. Due to the staggered timing of patient enrollment, the number of patients with data available decreases at later time points; these patients are still participating in the study.

Efficacy and PD (CK and Hex4) analyses were conducted in the efficacy population (patients who took at least one dose of 20 mg/kg cipaglucosidase alfa + 260 mg miglustat co-administration in stage 3 and had both a baseline and at least one post-baseline assessment for any efficacy endpoint). Efficacy and pharmacodynamic analyses are presented for pooled ERT-experienced ambulatory patients (i.e., cohort 1 and cohort 4), as the baseline demographics for patients in these cohorts are similar except for the duration of prior ERT, and separately for ERT-naïve ambulatory patients (cohort 3) and ERT-experienced non-ambulatory patients (cohort 2). Pharmacokinetics (plasma total GAA protein and plasma miglustat) are characterized using descriptive statistics. Full details are given in Supplementary Sect. S2. Safety evaluations were conducted on the safety population (patients who were exposed to at least one dose of the study drug; for stages 3 and 4, this included all cohort 1 patients who entered stage 3 and received at least one dose of 20 mg/kg cipaglucosidase alfa + 260 mg miglustat co-administration). Immunogenicity evaluations were conducted for patients in all cohorts, with blood samples collected at each visit before the infusion of the study drug. Further details are provided in Supplementary Sect. S1.

## Results

### Baseline demographics and disease characteristics

**Fig. 2:**
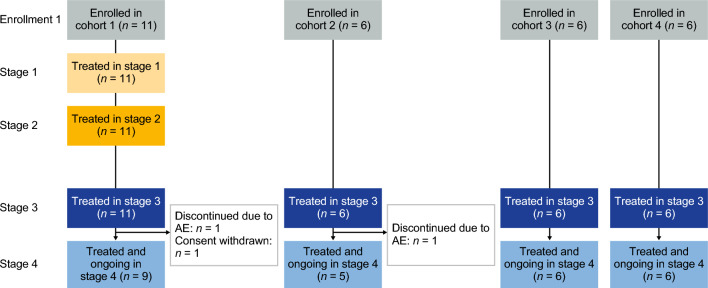
Patient disposition. Cohort 1: ERT experienced (2–6 years), ambulatory; cohort 2: ERT experienced (≥ 2 years), non-ambulatory; cohort 3: ERT naïve, ambulatory (1 patient in cohort 3 received a single dose of alglucosidase alfa before moving to Australia where this treatment is unavailable and was therefore eligible for inclusion in cohort 3); cohort 4: ERT experienced (≥ 7 years), ambulatory. *AE* adverse event, *ERT* enzyme replacement therapy

Twenty-nine patients were screened and enrolled from April 1, 2016, to June 7, 2019. Three patients (two in cohort 1 and one in cohort 2) discontinued the study during stage 3 (in the first 2 years of treatment with cipaglucosidase alfa + miglustat) before the data cutoff date of December 13, 2021 (Fig. 2). Mean (standard deviation [SD]) treatment duration during the study of the ERT-experienced patients was 51.8 (21.46), 46.3 (22.86) and 37.7 (4.13) months in cohorts 1, 2 and 4, respectively, and 54.7 (12.14) months for the ERT-naïve patients in cohort 3. Treatment compliance was high (mean ≥ 95.6%) for all stages of the study. Due to the staggered enrollment dates, some patients, who were still ongoing in the study, were yet to reach the 48-month timepoint at the data cutoff date.

Patient demographics at baseline were representative of the adult Pompe disease population [32, 33]. The mean (SD) duration of treatment with alglucosidase alfa at baseline was 7.8 (3.75) years in the ERT-experienced cohorts (cohorts 1, 2, and 4); all ERT-experienced patients received 20 mg/kg every 2 weeks before study enrollment except two patients in cohort 2 who received 40 mg/kg every 2 weeks. Mean baseline values for % predicted 6MWD were similar between cohorts 1 and 4 (61.0 and 59.0, respectively), and slightly numerically larger in cohort 3 (67.8). The mean MMT lower extremity score (hip and knee muscle groups) was generally similar across cohorts 1, 4, and 3 (Table 1). The mean % predicted sitting FVC was similar for cohorts 1 and 3 (52.6 and 57.2, respectively), higher in cohort 4 (65.3), and lower for the non-ambulatory ERT-experienced cohort 2 (42.3; Table 1).

**Table 1:** Baseline demographic and clinical characteristics (all enrolled patients)

	ERT experienced			ERT naïve
	Cohort 1	Cohort 2	Cohort 4	Cohort 3
	2–6 years prior ERT	non-ambulatory with ≥ 2 years prior ERT	≥ 7 years prior ERT	(*n* = 6)
	(*n* = 11)	(*n* = 6)	(*n* = 6)	
Age, years				
*n*	11	6	6	6
Mean (SD)	49.4 (9.5)	41.5 (18.1)	40.8 (17.0)	49.3 (15.1)
Median (range)	50.0 (28–66)	49.5 (18–57)	43.0 (20–65)	51.0 (24–65)
Sex, M:F	9:2	4:2	2:4	1:5
Race, *n* (%)				
White	8 (72.7)	3 (50.0)	5 (83.3)	1 (16.7)
Missing	3 (27.3)	3 (50.0)	1 (16.7)	5 (83.3)
Time since Pompe diagnosis, years				
*n*	11	6	6	6
Mean (SD)	7.7 (5.1)	15.9 (13.1)	13.0 (4.3)	5.2 (4.7)
Median (range)	6.3 (3–20)	12.5 (6–40)	12.8 (8–20)	3.7 (1–14)
Time on alglucosidase alfa, years				
*n*	11	6	6	N/A^a^
Mean (SD)	5.1 (1.3)	10.1 (4.8)	10.6 (2.1)	N/A^a^
Median (range)	6.3 (3–20)	10.0 (5–16)	10.1 (8–14)	N/A^a^
6MWD, % predicted				
*n*	10	N/A	6	6
Mean (SD)	61.0 (13.43)	N/A	59.0 (21.44)	67.8 (12.61)
Median (range)	62.8 (35–83)	N/A	64.6 (17–80)	67.0 (54–89)
6MWD, m				
*n*	10	N/A	6	6
Mean (SD)	397.2 (96.80)	N/A	387.3 (161.28)	396.0 (75.20)
Median (range)	394.5 (220–544)	N/A	431.0 (104–541)	395.2 (267–480)
Sitting FVC, % predicted				
*n*	10	3	6	6
Mean (SD)	52.6 (13.9)	42.3 (28.2)	65.3 (21.1)	57.2 (20.8)
Median (range)	53.0 (31–70)	39.0 (16–72)	66.0 (31–88)	59.0 (31–79)
MMT lower extremity score				
n	9	N/A	6	5
Mean (SD)	31.8 (1.9)	N/A	27.3 (3.7)	29.0 (1.7)
Median (range)	32.0 (30–34)	N/A	28.0 (21–30)	30.0 (26–30)

*6MWD* 6-min walking distance, *CFBL* change from baseline, *ERT* enzyme replacement therapy, *FVC* forced vital capacity, *MMT* manual muscle test, *N/A* not applicable, *SD* standard deviation

^a^*N* = 1 ERT-naïve patient received one dose of alglucosidase alfa > 6 months prior to study entry

### Efficacy outcomes: ambulatory patients (ERT-experienced pooled cohorts 1 + 4 and ERT-naïve cohort 3)

#### Motor function

**Fig. 3:**
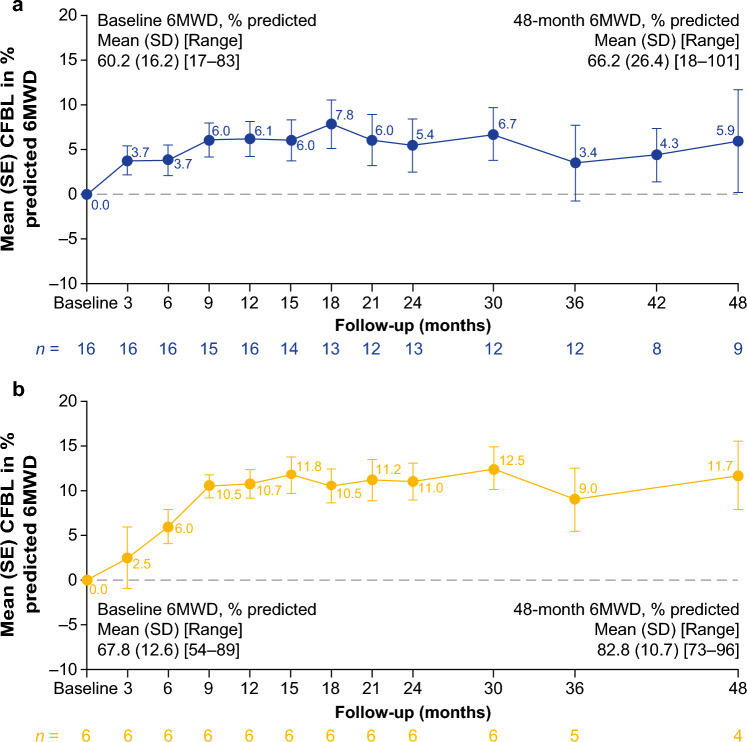
CFBL in % predicted 6MWD in ambulatory **a** ERT-experienced and **b** ERT-naïve patients. Pooled data from cohorts 1 and 4 for ERT-experienced patients. No applicable data are available for cohort 2 (non-ambulatory patients). *6MWD* 6-min walking distance, *CFBL* change from baseline, *ERT* enzyme replacement therapy, *SD* standard deviation, *SE* standard error

Both the ERT-experienced and ERT-naïve cohorts showed durable mean improvements from baseline in % predicted 6MWD up to 48 months (Fig. 3a and b; Supplementary Table S2), with 8/9 (88.9%) of ERT-experienced patients and 4/4 (100%) of ERT-naïve patients experiencing an improvement from baseline in % predicted 6MWD at month 48 (Supplementary Fig. S1). Absolute data for 6MWD in meters are summarized in Supplementary Table S2. After 12, 24, 36, and 48 months of follow-up, 6MWD in meters improved numerically from baseline in 13/16, 9/13, 6/12, and 6/9 ERT-experienced patients, respectively. After 12, 24, 36, and 48 months of follow-up, 6MWD in meters improved numerically from baseline in 6/6, 6/6, 4/5, and 4/4 ERT-naïve patients, respectively.

#### Respiratory function

**Fig. 4:**
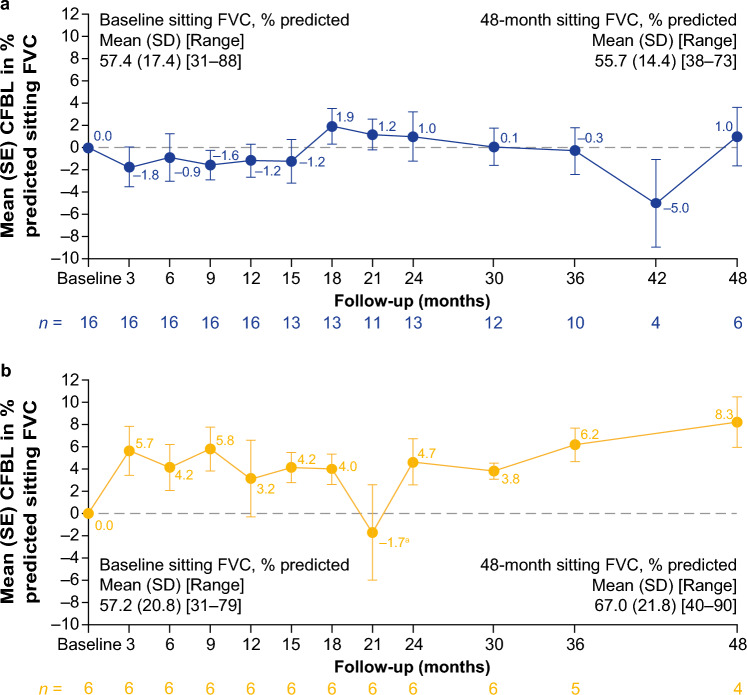
CFBL in % predicted FVC in ambulatory **a** ERT-experienced and **b** ERT-naïve patients. Pooled data from cohorts 1 and 4 for ERT-experienced patients. ^a^One patient in the ERT-naïve cohort experienced a large drop in % predicted FVC at month 21, which returned to previous levels at the following visit (month 24). *CFBL* change from baseline, *FVC* forced vital capacity, *SD* standard deviation, *SE* standard error

Mean CFBL in % predicted sitting FVC was generally stable (CFBL ± 3% points) for up to 48 months of follow-up in the ERT-experienced cohorts (Fig. 4a; Supplementary Table S2), with improvements (CFBL ≥ 3% points) at month 48 in 66.7% of ERT-experienced patients with available data (Supplementary Fig. S1). After 12, 24, 36, and 48 months of follow-up, % predicted sitting FVC improved (≥ 3% points) or remained stable (± 3% points) from baseline in 9/16, 11/13, 8/10, and 4/6 ERT-experienced patients, respectively. This is further supported by mean CFBL in % predicted maximum expiratory pressure (MEP) results that also improved numerically from baseline at 12, 24, 36, and 48 months of follow-up in ERT-experienced patients (Supplementary Table S2). Mean CFBL in % predicted maximum inspiratory pressure (MIP) initially improved numerically at 12 and 24 months of follow-up but declined numerically after 36 and 48 months of follow-up from baseline in ERT-experienced patients (Supplementary Table S2).

In the ERT-naïve cohort, mean CFBL in % predicted sitting FVC improved at 3 months and then remained generally stable up to 48 months of follow-up (Fig. 4b; Supplementary Table S2), except for a steep drop at month 21 where one patient experienced a large drop in % predicted sitting FVC, which returned to previous levels at the next visit at month 24. After 48 months of follow-up, 75% of ERT-naïve patients with available data showed improvements (≥ 3% points) in % predicted sitting FVC (Supplementary Fig. S1). While mean baseline values for % predicted sitting FVC in the ERT-naïve cohort (57.2%) and the ERT-experienced cohort (57.4%) were similar, mean improvements over 48 months of follow-up were more marked in the ERT-naïve cohort (Fig. 4b; Supplementary Table S2). After 12, 24, 36, and 48 months of follow-up, % predicted sitting FVC improved (≥ 3% points) or remained stable (± 3% points) from baseline in 5/6, 6/6, 5/5, and 4/4 in ERT-naïve patients, respectively. Percent predicted sitting FVC data were supported by similar outcomes in other pulmonary measures in ERT-naïve patients, including mean CFBL in % predicted MIP and MEP, which improved numerically at 12, 24, 36, and 48 months of follow-up (Supplementary Table S2).

#### 6MWD and % predicted sitting FVC clinically meaningful changes from baseline

At 48 months of follow-up, six ERT-experienced and three ERT-naïve ambulatory patients had available data for both 6MWD in meters and % predicted sitting FVC. In ERT-experienced patients, two demonstrated clinically meaningful improvements in both 6MWD and % predicted sitting FVC, four demonstrated clinically meaningful improvements or stabilization for at least one of 6MWD or % predicted sitting FVC, and none demonstrated clinically meaningful worsening for both 6MWD and % predicted sitting FVC. In ERT-naïve patients, one demonstrated clinically meaningful improvements in both 6MWD and % predicted sitting FVC, two demonstrated clinically meaningful improvements or stabilization for at least one of 6MWD or % predicted sitting FVC, and none demonstrated clinically meaningful worsening for both 6MWD and % predicted sitting FVC.

#### Muscle strength

**Fig. 5:**
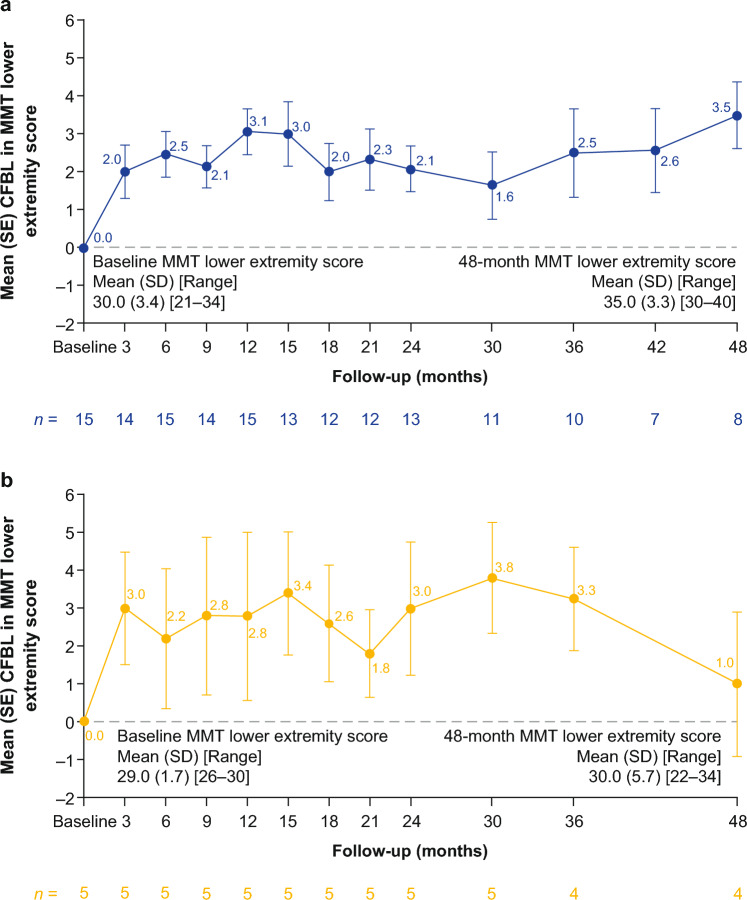
Absolute CFBL in MMT lower extremity score in ambulatory **a** ERT-experienced and **b** ERT-naïve patients. Pooled data from cohorts 1 and 4 for ERT-experienced patients. No applicable data are available for cohort 2 (non-ambulatory patients). *CFBL* change from baseline, *ERT* enzyme replacement therapy, *MMT* manual muscle test, *SD* standard deviation, *SE* standard error

Irrespective of ERT-treatment status at baseline, ambulatory patients experienced early improvements in muscle strength based on MMT lower extremity scores, which were maintained for up to 48 months of follow-up (Fig. 5; Supplementary Table S2). After 12, 24, 36, and 48 months of follow-up, the MMT lower extremity score improved numerically from baseline in 14/15, 11/13, 10/10, and 8/8 ERT-experienced patients and 4/5, 4/5, 4/4, and 3/4 ERT-naïve patients, respectively. MMT upper body scores (shoulder and elbow muscle groups) improved or stabilized from baseline in ambulatory ERT-experienced and ERT-naïve patients up to 48 months of follow-up (Supplementary Table S2).

#### PRO results

PROs supported the improvements observed for motor function, muscle strength, and pulmonary function tests. At baseline, all patients were significantly impacted by fatigue, which improved after 48 months of follow-up, as shown by the favorable mean CFBL in FSS. After 48 months of follow-up, all ambulatory patients reported stable R-PAct scores and RHS compared with baseline scores (Supplementary Table S2). SGIC outcomes for overall physical wellbeing improved in most patients across all cohorts after 48 months of follow-up (Supplementary Table S3). PGIC results indicated an improvement or stabilization for all cohorts after 48 months of follow-up and supported the results of other efficacy outcomes (Supplementary Table S3).

#### PD outcomes

**Fig. 6:**
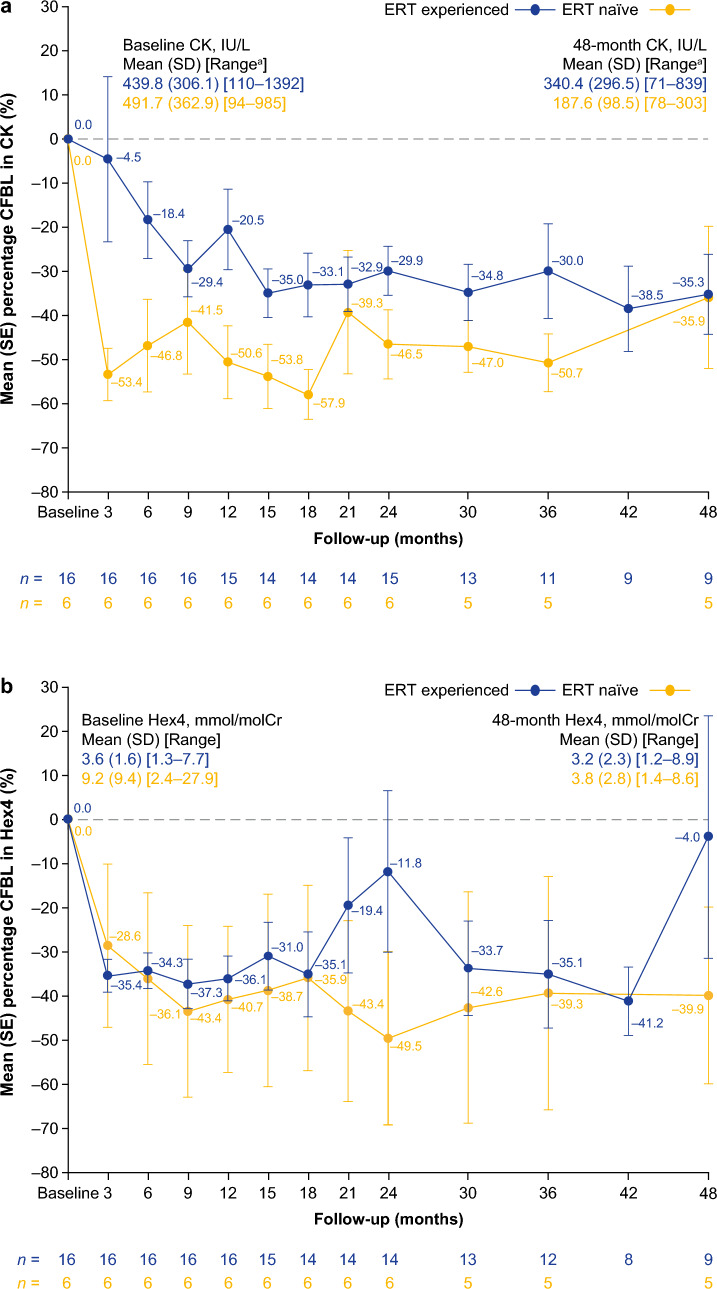
Percentage CFBL in **a** serum CK and **b** urine Hex4 in ambulatory patients over 48 months. Pooled data from cohorts 1, and 4 for ERT-experienced patients. ^a^Lower normal CK limit = 26 IU/L, upper normal CK limit = 192 IU/L. *CFBL* change from baseline, *CK* serum creatine kinase, *ERT* enzyme replacement therapy, *Hex4* urine glucose tetrasaccharide, *SD* standard deviation, *SE* standard error

During the 48 months of follow-up, cipa + mig was associated with mean reductions from baseline in plasma CK in both ERT-experienced and ERT-naive ambulatory cohorts (Fig. 6a; Supplementary Table S2). After 12, 24, 36, and 48 months of follow-up, plasma CK levels decreased numerically or remained stable from baseline in 13/15, 14/15, 9/11, and 8/9 ERT-experienced patients and 6/6, 6/6, 5/5, and 4/5 ERT-naïve patients, respectively (plasma CK levels increased in the remaining patients). Among patients who had CK data available at baseline and month 48, 6 of 9 (66.7%) ERT-experienced patients had abnormal CK levels at baseline and 2 of these (33.3%) had normal CK levels at month 48; 2 of 5 (40.0%) ERT-naïve patients had abnormal CK levels at baseline that remained abnormal at month 48. Overall, similar trends were observed with urine Hex4, which decreased numerically or remained stable from baseline after 12, 24, 36, and 48 months of follow-up in 16/16, 11/14, 11/12, and 6/9 ERT-experienced patients and 5/6, 5/6, 4/5, and 4/5 ERT-naïve patients, respectively (urine Hex4 levels increased in the remaining patients; Fig. 6b; Supplementary Table S2). In the ERT-experienced cohort, individual outlier values from different patients contributed to the peaks in mean Hex4 CFBL at months 21, 24, and 48, but these outliers did not affect the overall observed trends (Fig. 6b; Supplementary Table S2). Among patients who had Hex4 data available at baseline and at month 48, 5 out of 9 (55.6%) ERT-experienced patients had abnormal Hex4 levels at baseline, and 4 of these (80.0%) had normal Hex4 levels at month 48; 4 out of 5 (80.0%) ERT-naïve ambulatory patients had abnormal Hex4 measurements at baseline, and 1 of these (25.0%) returned to normal Hex4 levels at month 48, while the other 3 remained elevated.

### Efficacy outcomes: non-ambulatory ERT-experienced patients (cohort 2)

Limited long-term efficacy data are available for the non-ambulatory ERT-experienced cohort 2. Percent predicted sitting FVC data were available for 2 non-ambulatory ERT-experienced patients after 36 months and 1 patient at 48 months of follow-up. After 36 months of follow-up, 1 patient improved and the other worsened compared with baseline. The patient with available data after 48 months of follow-up was generally stable compared with baseline (Supplementary Table S2).

Non-ambulatory ERT-experienced patients showed durable mean percent reductions from baseline in plasma CK levels: 20.8% (*n* = 5), 25.3% (*n* = 5), 27.1% (*n* = 3), and 23.7% (*n* = 2) at 12, 24, 36, and 48 months, respectively. In addition, a consistent decrease was also seen in urine Hex4 levels from baseline over the follow-up period. After 12, 24, 36, and 48 months, the mean percent decrease from baseline in Hex4 levels were 15.6% (*n* = 5), 34.1% (*n* = 5), 36.5% (*n* = 4), and 9.4% (*n* = 2), respectively (Supplementary Table S2).

PRO results are available for 2 non-ambulatory ERT-experienced patients up to 48 months of follow-up and demonstrate improved FSS and stable RHS scores compared with baseline. R-PAct scores also showed a mean improvement from baseline up to 48 months of follow-up in the 2 non-ambulatory ERT-experienced patients, indicating an improved ability to perform daily activities and participate in social situations (Supplementary Table S2). One patient reported no change, and 1 reported an improvement from baseline in SGIC’s overall physical wellbeing. The PGIC results for the 2 ERT-experienced non-ambulatory patients showed no change for 1 patient and a decline for the other (Supplementary Table S3).

### Pharmacokinetic outcomes

Blood sampling for plasma GAA protein and miglustat concentrations was taken for cohort 1 (ERT experienced) and cohort 3 (ERT naïve) only. Blood sampling for PK was not performed for cohort 2 (non-ambulatory ERT-experienced) or cohort 4 (ERT-experienced) due to the expected similarity in PK assessments to cohort 1 and to prevent any undue burden on patients. Plasma total GAA protein, measured by signature peptide T09, increased dose-dependently after single ascending doses of 5 mg/kg, 10 mg/kg and 20 mg/kg cipaglucosidase alfa (Supplementary Fig. S2). Generally, the median time of peak signature peptide T09 concentrations (*t*_max_) was similar across all treatments and consistent with the duration of cipaglucosidase infusion of approximately 4 h. Exposures (*C*_max_ and the area under the plasma drug concentration–time curve [AUC]) increased dose-dependently. Plasma total GAA protein exposures were similar between cohort 1 and cohort 3 ambulatory patients. Plasma miglustat exposures increased dose-dependently in cohort 1 ambulatory patients. PK outcomes are summarized in Supplementary Tables S4 and S5.

Plasma total GAA protein by signature peptide T09 AUCs were estimated by population PK analysis (median: 1700 µg·h/mL, range: 1520 to 1870 µg·h/mL) at 24 months from cohorts 1 and 3. The data were similar to AUCs estimated by noncompartmental analysis (median: 1796 µg·h/mL, range: 1240 to 2652 µg·h/mL) following the first and third doses at 20 mg/kg cipaglucosidase alfa + 260 mg miglustat from cohort 1 and cohort 3 (Supplementary Fig. S3).

Administration of miglustat 1 h before cipaglucosidase alfa infusion resulted in greater AUCs than 20 mg/kg cipaglucosidase alfa alone (Supplementary Fig. S2). The distribution phase half-life was increased by 26.7% and 47.7% following 130 mg and 260 mg miglustat co-administration, respectively (mean t1/2α [CV%, percentage coefficient of variation]: cipaglucosidase alfa 20 mg/kg, 1.52 h [9.2]; cipaglucosidase alfa 20 mg/kg + miglustat 130 mg first dose, 1.9 h [10.6]; cipaglucosidase alfa 20 mg/kg + miglustat 260 mg first dose, 2.2 h [19.1]; Supplementary Table S4). Miglustat was rapidly cleared from circulation.

### Safety

Cipa + mig was generally well tolerated; safety outcomes are shown in Table 2. The most frequently reported treatment-emergent adverse events (TEAEs; > 40% of patients) for the overall population were falls, nasopharyngitis, diarrhea, headache, and arthralgia. Most TEAEs were mild or moderate in severity. TEAEs leading to study withdrawal occurred in 2 patients: 1 patient in cohort 1 had diffuse large B-cell lymphoma, which the investigator assessed as unrelated to treatment, and 1 patient in cohort 2 had a drug-related TEAE of urticaria, considered to be an IAR. The incidence of IARs was similar between ERT-experienced (48%) and ERT-naïve (50%) cohorts. Most patients who experienced IARs responded well to pre-infusion medications given to mitigate IARs and continued on study treatment.

**Table 2:** Summary of adverse events

	ERT experienced^a^ (*n* = 23)	ERT naïve (*n* = 6)	Overall (*N* = 29)
Patients with any TEAE, *n* (%)	23 (100)	6 (100)	29 (100)
TEAEs potentially related to treatment	16 (70)	4 (67)	20 (69)
Serious TEAEs	8 (35)	4 (67)	12 (41)
Serious TEAEs potentially related to treatment	2 (9)	2 (33)	4 (14)
TEAEs leading to study withdrawal	2 (9)	0 (0)	2 (7)
Severe TEAEs	6 (26)	3 (50)	9 (31)
TEAEs leading to death	0 (0)	0 (0)	0 (0)
IARs	10 (43)	3 (50)	13 (45)

*ERT* enzyme replacement therapy, *IAR* infusion-associated reaction, *TEAE* treatment-emergent adverse event with onset date on or after the first dose of study drug

^a^Pooled data for ERT-experienced cohorts 1, 2 and 4 (includes both ambulatory and non-ambulatory patients)

### Immunogenicity outcomes

Immunogenicity data up to the data cutoff date of December 13, 2021, are presented. The mean (SD) duration of cipa + mig treatment was 48.4 (18.04) months for patients tested for ADAs at the data cutoff date. At baseline, ADAs were present in 21/23 ERT-experienced patients (median titer 12,800 [range 200–1,638,400]) and 1/6 ERT-naïve patients (titer 400), based on a cutoff established using 75 ERT-naïve human plasma samples (Supplementary Section S1). In patients with available data after 48 months of follow-up, the median (range) ADA titer was 9600 (1600–1,638,400; *n* = 9) for ERT-experienced patients and 3200 (800–1,638,400; *n* = 5) for ERT-naïve patients. After treatment with cipa + mig, ADAs were present in all patients, meaning that 2 ERT-experienced patients seroconverted with a median peak titer of 1,664,000 (interquartile range [IQR] calculated as the difference between the upper and lower quartiles [Q3–Q1], 1,612,800) and 5 ERT-naïve patients seroconverted, with a median peak titer of 102,400 (IQR, 51,200). In ERT-experienced patients with ADAs at baseline, titers increased by ≥ fourfold in 17 patients with a median peak titer of 102,400 (IQR, 102,400). In the ERT-naïve patient with ADAs at baseline, the peak titer was 3,276,800 after treatment.

Most ERT-experienced patients (22, 95.7%) and all ERT-naïve patients tested positive for at least one type of NAb at intermittent time points (Supplementary Section S1). At baseline, 12 ERT-experienced patients and no ERT-naïve patients tested positive for antibodies cross-reactive to alglucosidase alfa. After treatment, 17 ERT-experienced patients and 5 ERT-naïve patients were positive for antibodies cross-reactive to alglucosidase alfa.

Anti-rhGAA IgE antibodies were measured at baseline for 21 of 23 ERT-experienced patients and all 6 ERT-naïve patients and after treatment only if anaphylaxis or a moderate to severe IAR occurred, as determined by the study investigator. One ERT-experienced patient and none of the ERT-naïve patients were positive for anti-rhGAA IgE antibodies at baseline. Six patients were tested for anti-rhGAA IgE antibodies post-treatment (after an IAR), and 1 ERT-naïve patient tested positive.

## Discussion

The therapeutic limitations of treatment for Pompe disease with alglucosidase alfa created an unmet need for new therapies to treat this debilitating disease. Long-term studies with alglucosidase alfa have shown that, despite initial improvement or stabilization, patients eventually experience a plateau or decline in muscle strength and motor and respiratory function [9, 10]. Additionally, the instability of alglucosidase alfa in the bloodstream and low bis-M6P N-glycan phosphorylation of alglucosidase alfa results in poor biodistribution and inefficient uptake by muscle cells [20]. Only a small amount of infused enzyme reaches the intended muscle tissue due to liver filtration, so efficient uptake of the remaining molecules is essential for maximal activity [20]. Novel treatments are needed to provide more efficient rhGAA delivery to muscle cells and offer patients durable and sustained improvements over the longer term [9–12].

Cipa + mig is a novel two-component therapy designed to overcome the challenges associated with alglucosidase alfa treatment [28, 29]. Cipaglucosidase alfa is enriched with bis-M6P *N*-glycans, which mediates more efficient delivery to skeletal muscle cells by improved CI-MPR uptake [20, 28, 31]. The CHO-derived cipaglucosidase alfa retains its ability to be fully processed into a mature form of the GAA enzyme within the target cell, thus maximizing catalytic activity [28, 31]. Combining cipaglucosidase alfa with the enzyme stabilizer miglustat enhances its stabilization in the bloodstream, improves biodistribution, and maintains the catalytic activity of cipaglucosidase alfa before muscle cell uptake [28, 29, 31]. Animal models support the proposed mode of action for cipa + mig. Cipa + mig improved multiple defects along the Pompe disease pathogenic cascade in GAA knock-out mice, including reduced lysosomal enlargement and autophagic build-up, resulting in improved muscle quality, architecture, and strength compared with alglucosidase alfa-treated or untreated mice [31, 34]. The potential for long-term benefits of cipa + mig was also suggested by early results of the Phase I/II ATB200-02 study [35] and the 12-month Phase III PROPEL study, in which clinically meaningful improvements were seen in crucial motor and respiratory functions for cipa + mig compared with alglucosidase alfa in patients with LOPD [32].

Data from the ATB200-02 study provide credible evidence for durable long-term improved outcomes with cipa + mig treatment. In ATB200-02, ambulatory ERT-experienced patients showed long-lasting mean improvements in 6MWD and maintained stable respiratory function up to 48 months of follow-up. Taken together, these results represent an encouraging improvement relative to the expected decline in motor and respiratory function experienced by many patients receiving long-term ERT [10].

Results for the ambulatory ERT-naïve population in ATB200-02 provide the first evidence for the long-term durability of the effect of cipa + mig on motor and respiratory function for up to 48 months of follow-up in previously untreated patients. The ongoing PROPEL long-term extension study will provide further opportunities to collect long-term data for treatment in this patient population [36].

Overall, stability in PROs (FSS, R-PAct, RHS) indicated a treatment benefit and supports the observed clinical outcomes of motor function, muscle strength, and pulmonary function in ERT-experienced and ERT-naïve ambulatory cohorts. Additionally, the SGIC and PGIC signaled an improvement or stabilization in ERT-experienced and ERT-naïve ambulatory cohorts after 48 months of follow-up. These results suggest that the positive clinical outcomes observed with cipa + mig treatment for up to 48 months translate into tangible benefits for ambulatory patients’ wellbeing and quality of life independent of their ERT status.

This is the first interventional study in adult patients with Pompe disease to include non-ambulatory, ERT-experienced patients. Due to the small sample size and because enrollment in this cohort only started in stage 3 of the study, more data are needed to determine the long-term efficacy in the non-ambulatory ERT-experienced cohort. Nevertheless, the durable reductions from baseline in plasma CK levels and consistent decrease in urine Hex4 levels from baseline over the 48-month follow-up period suggest the potential of cipa + mig as an effective treatment option in patients with severe disease burden.

Overall, the PD data support clinical outcome data. The persistent decreases seen in CK and Hex4 levels from baseline throughout the 48-month follow-up period in the current study for all cohorts provide adjunctive evidence for the benefits of cipa + mig. While these markers have not been clinically validated in LOPD, serum CK and urinary Hex4 are biomarkers of muscle damage and skeletal glycogen clearance, respectively, and are elevated in patients with Pompe disease [37, 38]. Correlations between improvements in these biomarkers and improvements in clinical outcomes have been demonstrated in a population of pediatric LOPD and IOPD patients [39]. Therefore, reductions of these elevated CK and Hex4 levels in ambulatory and non-ambulatory patients treated with cipa + mig in the current study may indicate a clinical benefit in adult LOPD.

The observed increase in cipaglucosidase alfa AUC with 260 mg miglustat relative to cipaglucosidase alfa alone is focused during the terminal elimination distribution phase, resulting in an approximate 47% increase in the alpha phase terminal half-life. This increase of partial AUC from t_max_ (end of infusion) to 24 h after the start of infusion observed with miglustat suggests that miglustat stabilizes cipaglucosidase alfa in the blood and therefore increases its availability for cellular uptake. This further indicates that adding miglustat may meaningfully contribute to the clinical response of cipaglucosidase alfa over the therapy duration.

The safety profile of cipa + mig was similar to that reported for alglucosidase alfa [16]. These safety data are consistent with observations in the PROPEL study in that cipa + mig was generally well tolerated with a favorable safety profile [32]. Additionally, no unexpected safety events were seen over the extended treatment period. Most TEAEs were mild or moderate in severity, and two TEAEs led to study withdrawal (one unrelated event of diffuse large B cell lymphoma and one related IAR of urticaria). Ongoing data collection from the PROPEL study open-label extension will further improve understanding of the long-term cipa + mig safety profile.

After 48 or more months of follow-up, ADAs were detected in all patients. Some ERT-experienced patients who tested positive for ADAs at baseline had peak titers that were higher than baseline during the study. At 48 months, the ERT-experienced median ADA titer was similar to the baseline, although the ADA titer range varied widely across patients. It is important to note that, due to differences in assay methodology between clinical trials, direct comparisons of the incidence or titers of ADAs in clinical trials of other rhGAA products are not possible. Most enrolled patients tested positive for NAbs throughout the 48-month follow-up period. Further studies are needed to investigate if an increase in ADA titers correlates with reduced efficacy or leads to safety concerns. At baseline, only 1 ERT-experienced patient from the 29 patients enrolled in the study tested positive for anti-rhGAA IgE antibodies. Of the 6 patients tested after treatment, only 1 ERT-naïve patient tested positive for anti-rhGAA IgE antibodies.

Given the progressive nature of Pompe disease [1, 6, 33] and long-term decline with standard-of-care treatment [9–12], the positive mobility and respiratory findings seen in the current study are encouraging. Worsening mobility necessitates wheelchair use, associated with a significant reduction in quality of life, greater disability, and greater burden on caregivers [9, 10, 40]. Respiratory failure and the need for ventilator support represents a second significant and negative milestone for patients with Pompe disease and is associated with an exceptionally high disease burden and a reduced quality of life [9, 10, 40]. While minimal clinically important differences for absolute 6MWD in meters and % predicted sitting FVC have not yet been established for LOPD, previous studies of other chronic diseases have identified an absolute CFBL in 6MWD change of 24–54 m (equivalent to a relative CFBL of 5–11%) and an absolute CFBL in % predicted sitting FVC change of 2–6% (equivalent to a relative CFBL of 3–9%) as clinically meaningful [40]. Our 48-month data, with a mean absolute CFBL of 30.4 m in ambulatory patients, along with improvements in muscle function, suggest that patients may be able to maintain their mobility over the long term with cipa + mig treatment. Additionally, our 48-month data, with a mean CFBL of 3.9% in sitting FVC in ambulatory patients, suggest a marked clinical benefit and the potential to delay the need for ventilatory assistance.

## Limitations

As might be expected for a phase I/II study of a rare disease therapy, the sample size was relatively small. Additionally, the heterogeneity between patients in each cohort may have introduced variability into the dataset due to the inherently variable nature of Pompe disease, which spans a broad spectrum of manifestations, disease severity, progression rates, and treatment responses. As the data set was relatively small, outcomes were only analyzed descriptively, with no statistical comparisons. Further data collection in the PROPEL long-term extension study will provide additional insight into the long-term outcomes of patients treated with cipa + mig [36].

## Conclusions

In this long-term (48-month) clinical trial, cipa + mig treatment showed sustained improvements or stabilization in motor and pulmonary function with a well-tolerated safety profile. When viewed alongside the phase III clinical data [32], the durable improvements shown in our study provide further evidence for the benefits of cipa + mig as a long-term treatment option to address the current unmet needs of patients with Pompe disease.

## Supplementary Information

Below is the link to the electronic supplementary material.

Supplementary file1 (PDF 839 KB)

Supplementary file2 (PDF 1954 KB)

## Data Availability

Data-sharing proposals and requests will be reviewed on a case-by-case basis. Requests for data should be addressed to Mitchell Goldman at mgoldman@amicusrx.com. Requests will be reviewed by a medical steering committee.
