# Correction: Long-term safety and efficacy of cipaglucosidase alfa plus miglustat in individuals living with Pompe disease: an open-label phase I/II study (ATB200-02)

**DOI:** 10.1007/s00415-025-12899-3

**Published:** 2025-02-17

**Authors:** Barry J. Byrne, Benedikt Schoser, Priya S. Kishnani, Drago Bratkovic, Paula R. Clemens, Ozlem Goker-Alpan, Xue Ming, Mark Roberts, Matthias Vorgerd, Kumaraswamy Sivakumar, Ans T. van der Ploeg, Mitchell Goldman, Jacquelyn Wright, Fred Holdbrook, Vipul Jain, Elfrida R. Benjamin, Franklin Johnson, Sheela Sitaraman Das, Yasmine Wasfi, Tahseen Mozaffar

**Affiliations:** 1 University of Florida, Gainesville, FL USA; 2 Friedrich-Baur-Institute at the Department of Neurology, LMU University Hospital, LMU Munich, Munich, Germany; 3 Duke University Medical Center, Durham, NC USA; 4 PARC Research Clinic, Royal Adelaide Hospital, Adelaide, SA Australia; 5 Department of Neurology, University of Pittsburgh School of Medicine and VA Pittsburgh Healthcare System, Pittsburgh, PA USA; 6 Lysosomal and Rare Disorders Research and Treatment Center, Fairfax, VA USA; 7 Neurology, Rutgers New Jersey Medical School, Newark, NJ USA; 8 Guam Regional Medical City, Dededo, Guam; 9 Salford Royal NHS Foundation Trust, Salford, UK; 10 Department of Neurology, University Hospital Bergmannsheil, Heimer Institute for Muscle Research, Bochum, Germany; 11 Neuromuscular Clinic and Research Center, Phoenix, AZ USA; 12 Erasmus MC University Medical Center, Rotterdam, Netherlands; 13 Amicus Therapeutics, Inc., Princeton, NJ USA; 14 University of California, Irvine, Irvine, CA USA

**Correction: Journal of Neurology (2023) 271:1787–1801** 10.1007/s00415-023-12096-0

In the original version of this article, the wrong Supplementary file 1 was originally published with this article and it has now been replaced with the correct file.

The original article has been corrected.

## Supplementary Information

Below is the link to the electronic supplementary material.

Supplementary file1 (PDF 839 kb)

