# Supplementary material for: Long-term safety and efficacy of cipaglucosidase alfa plus miglustat in individuals living with Pompe disease: an open-label phase I/II study (ATB200-02)
Source: J Neurol. 2023 Dec 6;271(4):1787–801. doi: 10.1007/s00415-023-12096-0 (PMC10973052; doi:10.1007/s00415-023-12096-0)
Supplement: Supplementary file 1 — Supplementary file1 (PDF 839 KB) [file 415_2023_12096_MOESM1_ESM.pdf]

Supplementary material

Long-term safety and efficacy of cipaglucosidase alfa plus miglustat in individuals living with Pompe disease: an open-label Phase I/II study (ATB200-02)

Journal name: *Journal of Neurology*

Barry J. Byrne,[1] Benedikt Schoser,[2] Priya S. Kishnani,[3] Drago Bratkovic,[4] Paula R. Clemens,[5] Ozlem Goker-Alpan,[6] Xue Ming,[7] Mark Roberts,[8] Matthias Vorgerd,[9] Kumaraswamy Sivakumar,[10] Ans T. van der Ploeg,[11] Mitchell Goldman[12], Jacquelyn Wright,[12] Fred Holdbrook,[12] Vipul Jain,[12] Elfrida R. Benjamin,[12] Franklin Johnson,[12] Sheela Sitaraman Das,[12] Yasmine Wasfi,[12] Tahseen Mozaffar[13]

Corresponding author: Dr. Barry J. Byrne; barry.byrne@ufl.edu

Supplementary Section S1: efficacy, pharmacodynamic (PD), safety, and immunogenicity assessments 3

Supplementary Section S2: pharmacokinetic (PK) analyses 6

Supplementary Table S1 Patient inclusion and exclusion criteria 7

Supplementary Table S2 Summary of clinical outcomes 9

Supplementary Table S3 Summary of Subject and Physician Global Impression of Change (S/PGIC) at 48 months follow-up 14

Supplementary Table S4 Summary of pharmacokinetic outcomes for plasma signature peptide T09 15

Supplementary Table S5 Summary of pharmacokinetic outcomes for plasma miglustat 17

Supplementary Fig. S1 Proportion of patients with CFBL in % predicted 6MWD for ambulatory (a) ERT-experienced and (b) ERT-naïve patients and CFBL in % predicted FVC for ambulatory (c) ERT-experienced and (d) ERT-naïve patients at month 48. 18

Supplementary Fig. S2 Plasma total GAA protein by signature peptide T09 concentration time profiles in ambulatory, ERT-experienced patients receiving cipaglucosidase alfa with and without miglustat. 19

Supplementary Fig. S3 Plasma total GAA protein by signature peptide T09 concentration AUCs estimated by population PK analysis at 24 months and estimated by non-compartmental analysis following the first and third doses of 20 mg/kg cipaglucosidase alfa + 260 mg miglustat in cohorts 1 and 3. 20

Supplementary Section S1: efficacy, pharmacodynamic (PD), safety, and immunogenicity assessments

For efficacy and PD outcomes, baseline was defined as the assessment on or prior to the first dose of any study medication (cipaglucosidase alfa alone in stage 1 or cipaglucosidase alfa plus miglustat [cipa+mig] in stages 2–4) for all cohorts. For safety, baseline was defined as the evaluation on or prior to the first dose of 20 mg/kg cipaglucosidase alfa plus 260 mg miglustat in stage 2 for cohort 1 and stage 3 for cohorts 2, 3, and 4. Efficacy, PD, safety, and immunogenicity outcomes assessed over 48 months are described below.

Motor function tests

Outcomes for motor function tests were summarized for baseline, every 3 months in stage 3, and every 6 months in stage 4 for all ambulatory patients (cohorts 1, 3, and 4). The actual score, absolute change, and % change from baseline (CFBL) for 6-minute walking distance (6MWD), and % predicted 6MWD were summarized using descriptive statistics for each scheduled visit. The following thresholds were used to characterize clinically meaningful relative CFBL for 6MWD in meters: ≥ 6% improving, ± 6% stability, and < −6% worsening. The time in seconds that the patient took to accomplish other motor tasks (i.e., Gowers' maneuver, 10-meter walk test [10MWT], 4-stair climb, chair test, and timed up and go [TUG]) were also summarized descriptively. The Gait, Stairs, Gowers' maneuver, and Chair (GSGC) score was analyzed as a continuous variable.

Pulmonary function tests (PFTs)

Sitting and supine forced vital capacity (FVC) and % predicted FVC, as well as maximum inspiratory pressure (MIP), maximum expiratory pressure (MEP), and sniff nasal inspiratory pressure (SNIP) were summarized at baseline, every 3 months in stage 3, and every 6 months in stage 4 for all ambulatory patients and for non-ambulatory patients without invasive ventilatory support. The actual value, absolute change, and % CFBL in each PFT at each scheduled visit were presented by cohort using descriptive statistics. The % predicted MIP, MEP, and SNIP were similarly summarized. The following thresholds were used to characterize clinically meaningful absolute CFBL in % predicted sitting FVC: ≥ 3% points improving, ± 3% points stability, and < −3% points worsening.

Muscle strength tests

Manual muscle tests (MMTs) were summarized at baseline, every 3 months in stage 3, and every 6 months in stage 4 for all patients. Ambulatory patients were asked to perform all muscle tests, while non-ambulatory patients were asked to perform only the upper body muscle tests. Muscle groups tested included shoulder abductors, shoulder adductors, elbow

extensors, elbow flexors, hip flexors, hip abductors, knee extensors and knee flexors. Body part (shoulder, elbow, knee, and hip), proximal muscles (shoulder and hip), body side (lower, upper) and total MMT (if scores were available for all applicable muscle groups) were recorded. The actual score and absolute CFBL in the MMT scores were presented by cohort using descriptive statistics.

Patient-reported outcomes (PROs)

PROs were assessed at baseline, every 3 months in stage 3 and every 6 months in stage 4. PROs assessed included the Rasch-built Pompe-specific Activity (R-PAct) scale, Rotterdam Handicap Scale (RHS) and Fatigue Severity Scale (FSS). Patients were given questionnaires to complete at scheduled visits before any study-related procedures took place.

Global Impression of Change

The Subject Global Impression of Change (SGIC) and Physician Global Impression of Change (PGIC) were only conducted during stages 3 and 4. The administrator of the SGIC read the questions to the patient.

PD parameters (biomarkers: serum creatine kinase [CK] and urine glucose tetrasaccharide [Hex4])

PD parameters (Hex4 and CK) were analyzed at every scheduled visit (except baseline) in stages 1 and 2, every 3 months in stage 3 and every 6 months in stage 4 and recorded as part of the safety laboratory assessment. The original study baseline at visit 2 was used as the baseline for PD parameters. Mean ± standard deviation (SD) at baseline, mean ± standard error (SE) of absolute CFBL, and mean ± standard error (SE) of percent CFBL were plotted for serum CK and urine Hex4.

Safety assessments

All treatment-emergent adverse events (TEAEs) were recorded and categorized as any TEAE or treatment-emergent serious adverse events (TESAEs); treatment-related TEAE or TESAEs; TEAEs or TESAEs leading to study drug discontinuation; TEAEs or TESAEs related to study drug and leading to study drug discontinuation; deaths. All adverse events (AEs) were defined as mild, moderate or severe based on medical judgment. TEAEs were defined as AEs that began after receiving the study medication in stage 2, period 5. Events that occurred > 30 days after the last dose of study drug were not considered treatment emergent. AEs with an unknown date of onset and a stop date after the start of the study period or unknown were included as TEAEs. Any AE with a start date equal to the date of first dose, where the start of the AE could not be definitively placed prior to the first dose, was considered treatment

emergent. The onset of a new illness and any pre-existing complaint or symptom that worsened after the patients received the first dose of the study drug was recorded as a TEAE.

Infusion-associated reactions (IARs) were classified into immediate (AE onset up to 24 hours after infusion) or late subtypes (AE onset between 24 hours and 3 days after infusion) and divided into five severity grades (mild, moderate, severe, life-threatening and lethal).

Immunogenicity assessments

Blood samples for immunogenicity assessments were collected from all patients at each visit prior to infusion of the study drug. Samples were first tested for anti-cipaglucosidase alfa (drug) antibodies (ADAs) in the total ADA assay. The ADA assessment used a novel electrochemiluminescence-based immunoassay and Meso Scale Discovery bridging format (Meso Scale Diagnostics, MD, USA). A validated screening assay cut-off was established using 75 ERT-naïve human plasma samples (BioAgilytix Labs, Durham NC, USA). The assay sensitivity was ≤ 100 ng/mL (screening assay: 8.07 ng/mL), and the minimum required dilution was 100-fold. Screened positive samples were assessed in a confirmatory assay via competition with unlabeled cipaglucosidase alfa. The confirmatory assay sensitivity was determined to be 18.4 ng/mL. It is important to note that due to differences in assay methods between clinical trials, direct comparisons of these data to the incidence or titers of ADAs in clinical trials of other rhGAA products are not possible.

Only samples that tested positive and confirmed for ADAs were further tested for ADA titers, as well as neutralizing antibodies (NAbs) and antibodies cross-reactive to alglucosidase alfa, as per the following:

- Inhibition of recombinant human GAA (rhGAA)-mediated hydrolysis of 4-MU-α-Glc (4-MU-α-Glc NAb)
- Inhibition of rhGAA-mediated hydrolysis of glycogen (glycogen NAb)
- Inhibition of rhGAA binding to cation-independent mannose-6-phosphate receptor ([CI-MPR] NAb)
- Only positive specific antibodies cross-reactive to alglucosidase alfa were assessed in the cross-reactivity assay (no titers were assessed).

Anti-rhGAA-specific immunoglobulin E (IgE) antibodies were assessed at baseline and after the occurrence of anaphylaxis or a moderate or severe IAR as determined by the study investigator.

Supplementary Section S2: pharmacokinetic (PK) analyses

Serial blood samples were collected for 24 hours following single ascending doses of cipaglucosidase alfa alone (5 mg/kg, 10 mg/kg, and 20 mg/kg) for plasma total GAA protein concentrations in stage 1, for single and repeated doses of cipaglucosidase alfa 20 mg/kg with miglustat at ascending doses (130 mg and 260 mg) for both plasma total GAA protein concentrations and plasma miglustat concentrations in stage 2, and for single and repeated doses cipaglucosidase alfa 20 mg/kg with miglustat 260 mg for both plasma total GAA protein concentrations and plasma miglustat concentrations in ERT-naïve patients from cohort 3 during stage 3. Descriptive statistics were used to summarize plasma total GAA (based on cipaglucosidase alfa signature peptides T09 and T50) and plasma miglustat, both as measured by the liquid chromatography with tandem mass spectrometry method at each time point, including n, mean, SD, percentage coefficient of variation (CV%), median, minimum, and maximum. Geometric means (GeoMean) and CV% of GeoMean were calculated for maximum observed plasma concentration (C_{max}), the area under the plasma drug concentration–time curve (AUC) from time zero to observed time (t) (AUC_{0-t}), AUC from time zero to time infinity ($AUC_{0-\infty}$), partial AUC from t_{max} to 24 hours ($AUC_{tmax-24\ h}$); cipaglucosidase alfa only), alpha terminal half-life ($t_{1/2\alpha}$; cipaglucosidase alfa only), beta terminal half-life ($t_{1/2\beta}$), plasma clearance (CL_T for cipaglucosidase alfa and CL/F for miglustat), and volume of distribution (V_z/F, miglustat only). An analysis of variance (ANOVA) model was used to assess the relative bioavailability of plasma total GAA protein by signature peptide T09 with and without co-administration of miglustat. The natural log-transformed plasma total GAA protein by signature peptide T09 C_{max} and AUC_{0-t} was analyzed using a fixed-effects ANOVA model with term for the treatment.

Supplementary Table S1 Patient inclusion and exclusion criteria

Inclusion criteria	Cohort 1	Cohort 2	Cohort 3	Cohort 4
Male and female patients between 18 and 65 years of age, inclusive	X	X	X	X
Patient must provide signed informed consent prior to any study-related procedures	X	X	X	X
Patients of childbearing potential must agree to use medically accepted methods of contraception during the study and for 90 days after last co-administration of ATB200 and AT2221	X	X	X	X
Patient has a diagnosis of Pompe disease based on documented deficiency of GAA enzyme activity or by *GAA* genotyping	X	X	X	X
Patient has received ERT with alglucosidase alfa (Myozyme/Lumizyme) for the previous 2 to 6 years, inclusive	X			
Patient has received ERT with alglucosidase alfa (Myozyme/Lumizyme) for ≥ 2 years		X		
Patient has received ERT for the previous ≥ 7 years				X
Patient is currently receiving alglucosidase alfa (Myozyme/Lumizyme) at a frequency of once every other week	X			X
Patient is currently receiving alglucosidase alfa (Myozyme/Lumizyme) at a regular or set frequency		X		
Patient has received and completed the last two infusions without a drug-related adverse event resulting in dose interruption	X	X		X
Patient must be able to walk between 200 and 500 meters on the 6MWT	X		X	
Patient must be able to walk between 75 and 600 meters on the 6MWT				X
Patient has documented 6MWT on three separate occasions, each at least 6 months apart with at least two values in the past 3 years				X
Upright FVC must be 30% to 80% of predicted normal value	X		X	
Upright FVC must be 30% to 85% of predicted normal value				X
Patient must be wheelchair-bound and unable to walk unassisted		X		

Exclusion criteria	Cohort 1	Cohort 2	Cohort 3	Cohort 4
Patient has received any investigational therapy including adjunctive therapy for Pompe disease, other than alglucosidase alfa within 30 days or 5 half-lives of the therapy or treatment, whichever is longer, prior to the baseline visit, or anticipates doing so during the study	X	X		X
Patient has received any ERT, including alglucosidase alfa at any time, or any investigational therapy for Pompe disease within 30 days or 5 half-lives of the therapy or treatment, whichever is longer, prior to the baseline visit, or anticipates doing so during the study			X	
Patient has received treatment with prohibited medications within 30 days of the baseline visit	X	X	X	X
Patient, if female, is pregnant or breastfeeding at screening	X	X	X	X
Patient, whether male or female, is planning to conceive a child during the study	X	X	X	X
Patient requires invasive ventilatory support	X		X	X
Patient uses non-invasive ventilatory support ≥ 6 hours a day while awake	X		X	X
Patient has a medical or any other extenuating condition or circumstance that may, in the opinion of the investigator or the medical monitor, pose an undue safety risk to the patient or compromise his/her ability to comply with protocol requirements	X	X	X	X
Patient has a history of anaphylaxis to alglucosidase alfa	X	X		X
Patient has a history of high sustained anti-rhGAA antibodies	X	X		X
Patient has a history of allergy or sensitivity to miglustat or other iminosugars	X	X	X	X
Patients with active systemic autoimmune disease such as lupus, scleroderma, or rheumatoid arthritis; all patients with autoimmune disease must be discussed with the Amicus Medical Monitor	X	X	X	X
Patients with active bronchial asthma; all patients with bronchial asthma must be discussed with the Amicus medical monitor	X	X	X	X

6MWT 6-minute walk test; *AE* adverse event; *ERT* enzyme replacement therapy; *GAA* acid α-glucosidase; *FVC* forced vital capacity; *rhGAA* recombinant human GAA

Supplementary Table S2 Summary of clinical outcomes

Outcome measure	Ambulatory patients								Non−ambulatory patients			
	ERT experienced				ERT naïve				ERT experienced			
**Timepoint (months)**	12	24	36	48	12	24	36	48	12	24	36	48
**Motor function**									N/A	N/A	N/A	N/A
Percent predicted 6MWD									N/A	N/A	N/A	N/A
n	16	13	12	9	6	6	5	4				
CFBL mean (SD)	6.1 (7.84)	5.4 (10.56)	3.4 (14.66)	5.9 (17.36)	10.7 (3.93)	11.0 (5.06)	9.0 (7.98)	11.7 (7.69)				
CFBL median (Q1, Q3)	4.2 (2.0, 10.1)	3.7 (0.6, 11.4)	3.1 (−0.5, 11.6)	8.8 (1.5, 15.6)	10.5 (7.6, 13.8)	10.0 (7.8, 14.6)	9.2 (7.8, 15.7)	10.3 (5.7, 17.8)				
6MWD, meters									N/A	N/A	N/A	N/A
n	16	13	12	9	6	6	5	4				
CFBL mean (SD)	33.5 (49.62)	25.2 (63.30)	9.8 (85.98)	20.7 (101.94)	57.0 (29.96)	54.4 (36.18)	43.5 (45.19)	52.2 (46.59)				
CFBL median (Q1, Q3)	20.6 (8.3, 51.9)	18.5 (−7.0, 58.0)	11.6 (−13.7, 51.5)	27.0 (−3.3, 83.7)	55.6 (30.8, 78.9)	49.0 (22.6, 78.2)	59.0 (20.5, 80.0)	40.5 (16.3, 88.0)				
10−meter walk test, seconds									N/A	N/A	N/A	N/A
n	16	14	12	9	6	6	5	5				
CFBL mean (SD)	−0.1 (1.47)	1.0 (3.19)	3.9 (12.56)	0.6 (1.74)	−0.9 (0.75)	−0.5 (1.00)	0.1 (0.61)	0.7 (1.66)				
CFBL median (Q1, Q3)	−0.5 (−1.1, 0.7)	0.3 (0.0, 1.7)	0.1 (−0.5, 1.5)	0.5 (−0.1, 1.5)	−0.9 (−1.5, −0.6)	−0.4 (−1.7, 0.5)	0.2 (−0.1, 0.4)	0.3 (−0.2, 1.1)				
Gait, Stairs, Gowers, and Chair									N/A	N/A	N/A	N/A

n	16	14	12	9	6	6	5	5				
CFBL mean (SD)	−1.3 (4.39)	−1.6 (5.20)	−1.1 (5.18)	−1.0 (6.02)	−0.8 (2.23)	−1.7 (2.07)	−2.0 (2.12)	−2.4 (2.70)				
CFBL median (Q1, Q3)	−0.5 (−5.0, 0.5)	−1.0 (−6.0, 0.0)	−0.5 (−5.5, 0.5)	−1.0 (−6.0, 0.0)	−0.5 (−1.0, 1.0)	−1.5 (−4.0, 0.0)	−2.0 (−3.0, 0.0)	−3.0 (−4.0, −2.0)				
Timed Up and Go, seconds												
n	15	13	12	9	6	6	5	5				
CFBL mean (SD)	−1.5 (2.68)	0.1 (2.24)	−1.5 (3.68)	−0.6 (2.41)	−0.7 (1.95)	−1.0 (2.00)	0.5 (3.59)	0.3 (2.33)	N/A	N/A	N/A	N/A
CFBL median (Q1, Q3)	−0.9 (−2.5, 0.3)	0.3 (−1.4, 0.7)	−1.2 (−3.7, 0.7)	−0.5 (−2.2, 0.6)	−1.0 (−2.1, −0.1)	−1.3 (−2.7, −0.5)	−0.5 (−1.7, 0.8)	−1.1 (−1.1, 1.0)				
**Pulmonary function**												
Percent predicted sitting FVC												
n	16	13	10	6	6	6	5	4	2	1	2	1
CFBL mean (SD)	−1.2 (5.95)	1.0 (7.96)	−0.3 (6.68)	1.0 (6.42)	3.2 (8.42)	4.7 (5.09)	6.2 (3.35)	8.3 (4.50)	2.5 (9.19)	2.0 (N/A)	−2.0 (7.07)	−1.0 (N/A)
CFBL median (Q1, Q3)	−1.0 (−5.5, 4.0)	1.0 (−2.0, 6.0)	−1.5 (−3.0, 2.0)	3.5 (−7.0, 5.0)	4.5 (−4.0, 11.0)	4.0 (0.0, 9.0)	5.0 (4.0, 6.0)	9.5 (5.0, 11.5)	2.5 (−4.0, 9.0)	2.0 (2.0, 2.0)	−2.0 (−7.0, 3.0)	−1.0 (−1.0, −1.0)
Percent predicted maximum inspiratory pressure %												
n	16	13	11	6	6	6	5	4	3	2	3	2
CFBL mean (SD)	3.7 (11.61)	4.5 (20.67)	−0.5 (22.23)	−3.8 (5.41)	8.0 (15.46)	18.7 (23.90)	12.5 (20.71)	16.1 (17.23)	1.1 (4.71)	3.3 (1.39)	−0.4 (5.20)	3.9 (2.35)
CFBL median (Q1, Q3)	0.2 (−1.2, 4.2)	−0.7 (−6.5, 7.1)	−5.0 (−6.7, −0.6)	−4.9 (−6.0, −1.5)	6.8 (−4.0, 10.4)	23.5 (−2.9, 32.3)	18.1 (−1.8, 18.5)	14.4 (3.6, 28.5)	0.1 (−3.0, 6.3)	3.3 (2.3, 4.2)	0.1 (−5.9, 4.4)	3.9 (2.3, 5.6)

Percent predicted maximum expiratory pressure %												
n	16	13	11	6	6	6	5	4	3	2	3	2
CFBL mean (SD)	9.4 (21.37)	16.8 (21.88)	13.7 (26.79)	17.9 (30.81)	11.6 (18.16)	17.9 (16.25)	14.2 (20.27)	24.8 (24.15)	10.6 (10.28)	21.1 (25.72)	11.5 (22.30)	21.0 (27.00)
CFBL median (Q1, Q3)	6.1 (−2.6, 16.4)	6.1 (0.9, 31.7)	7.1 (−3.5, 19.8)	8.1 (3.3, 41.2)	10.5 (−3.1, 16.4)	14.6 (6.3, 26.1)	5.2 (1.1, 16.0)	19.1 (8.5, 41.0)	9.2 (1.1, 21.5)	21.1 (2.9, 39.3)	−0.7 (−2.0, 37.3)	21.0 (2.0, 40.1)
Percent predicted Sniff Nasal Inspiratory Pressure %												
n	16	13	11	6	6	6	5	4	3	2	3	2
CFBL mean (SD)	2.1 (18.56)	5.3 (23.86)	−2.3 (23.65)	7.3 (21.79)	9.4 (13.04)	8.1 (11.48)	4.3 (9.30)	4.6 (7.76)	4.5 (13.96)	7.8 (13.65)	−0.4 (4.03)	−4.1 (3.26)
CFBL median (Q1, Q3)	−1.5 (−8.0, 11.4)	3.7 (−3.4, 13.8)	−2.5 (−3.5, 5.6)	−0.1 (−3.4, 28.1)	10.6 (−2.2, 15.8)	11.4 (−0.8, 15.9)	6.7 (−0.7, 9.2)	5.6 (−1.2, 10.4)	0.0 (−6.7, 20.1)	7.8 (−1.9, 17.4)	0.4 (−4.8, 3.1)	−4.1 (−6.4, −1.8)
**Muscle strength**												
Lower body MMT score												
n	15	13	10	8	5	5	4	4	N/A	N/A	N/A	N/A
Absolute CFBL mean (SD)	3.1 (2.34)	2.1 (2.18)	2.5 (3.69)	3.5 (2.51)	2.8 (4.97)	3.0 (3.94)	3.3 (2.75)	1.0 (3.83)	N/A	N/A	N/A	N/A
CFBL median (Q1, Q3)	2.0 (2.0, 5.0)	2.0 (1.0, 4.0)	1.5 (0.0, 4.0)	3.0 (2.0, 5.0)	4.0 (0.0, 5.0)	3.0 (2.0, 3.0)	3.5 (1.0, 5.5)	2.0 (−2.0, 4.0)				
Upper body MMT score												
n	15	13	11	8	6	6	5	5	4	4	4	N/A
Absolute CFBL mean (SD)	1.3 (2.52)	0.5 (2.30)	0.8 (2.75)	0.5 (2.00)	0.0 (1.67)	−1.5 (2.17)	1.0 (1.87)	−1.4 (2.61)	1.3 (3.40)	2.0 (6.63)	−0.8 (10.72)	N/A

CFBL median (Q1, Q3)	1.0 (0.0, 2.0)	0.0 (−1.0, 2.0)	0.0 (−2.0, 3.0)	0.0 (0.0, 1.5)	−0.5 (−1.0, 2.0)	−1.0 (−4.0, 0.0)	1.0 (0.0, 1.0)	−2.0 (−2.0, 0.0)	2.0 (−1.5, 4.0)	0.0 (−3.0, 7.0)	−1.0 (−9.0, 7.5)	N/A
Total MMT score												
n	15	13	10	8	5	5	4	4	4	4	4	N/A
Absolute CFBL mean (SD)	4.3 (3.20)	2.5 (3.45)	3.6 (5.23)	4.0 (3.70)	2.4 (4.39)	2.0 (5.70)	3.5 (3.00)	−1.3 (4.99)	1.3 (3.40)	2.0 (6.63)	−0.8 (10.72)	N/A
CFBL median (Q1, Q3)	4.0 (3.0, 6.0)	4.0 (0.0, 5.0)	3.5 (0.0, 6.0)	3.5 (1.5, 6.5)	3.0 (−2.0, 5.0)	2.0 (1.0, 3.0)	3.0 (1.0, 6.0)	−1.5 (−5.5, 3.0)	2.0 (−1.5, 4.0)	0.0 (−3.0, 7.0)	−1.0 (−9.0, 7.5)	N/A
**Patient−reported outcomes**												
Rasch−built Pompe−specific Activity												
n	15	13	11	9	6	6	5	5	5	4	4	2
CFBL mean (SD)	1.3 (2.97)	2.2 (2.59)	−0.1 (3.24)	−0.3 (2.96)	2.0 (3.46)	2.2 (3.43)	1.4 (1.52)	−0.6 (5.22)	1.4 (1.95)	0.8 (0.96)	0.8 (0.96)	2.0 (4.24)
CFBL median (Q1, Q3)	1.0 (−1.0, 5.0)	3.0 (0.0, 3.0)	0.0 (−2.0, 1.0)	−1.0 (−1.0, 0.0)	1.5 (−1.0, 3.0)	2.5 (−1.0, 4.0)	1.0 (0.0, 3.0)	0.0 (−1.0, 2.0)	0.0 (0.0, 3.0)	0.5 (0.0, 1.5)	0.5 (0.0, 1.5)	2.0 (−1.0, 5.0)
Rotterdam Handicap Scale												
n	15	14	11	9	6	6	5	5	4	4	4	2
CFBL mean (SD)	−0.3 (3.01)	−1.1 (3.91)	−2.3 (4.44)	−2.3 (3.16)	−0.4 (2.06)	−0.4 (2.14)	−1.2 (0.84)	−1.3 (2.56)	0.6 (3.15)	0.6 (4.66)	0.6 (1.67)	−1.5 (4.95)
CFBL median (Q1, Q3)	−1.0 (−3.0, 1.0)	−0.5 (−3.0, 2.0)	−2.0 (−5.0, 1.0)	−2.0 (−5.0, 0.0)	−0.1 (−1.0, 1.0)	−1.5 (−2.0, 2.0)	−1.0 (−2.0, −1.0)	−2.0 (−3.0, −1.0)	1.5 (−1.4, 2.7)	−0.6 (−2.8, 4.0)	1.3 (−0.4, 1.7)	−1.5 (−5.0, 2.0)
Fatigue Severity Scale												
n	15	14	11	9	6	6	5	5	5	5	5	2

CFBL mean (SD)	−2.9 (10.55)	−3.1 (8.82)	1.4 (16.74)	−3.4 (10.10)	−6.3 (7.06)	−5.5 (11.40)	−6.2 (10.57)	1.2 (7.95)	−9.2 (11.37)	−23.0 (17.78)	−10.0 (9.35)	−12.0 (15.56)
CFBL median (Q1, Q3)	−2.0 (−11.0, 4.0)	−3.0 (−7.0, 0.0)	0.0 (−10.0, 7.0)	−5.0 (−10.0, −3.0)	−3.0 (−12.0, −2.0)	−5.0 (−13.0, 3.0)	−11.0 (−12.0, 5.0)	4.0 (0.0, 6.0)	−13.0 (−15.0, 1.0)	−24.0 (−24.0, −16.0)	−15.0 (−16.0, −2.0)	−12.0 (−23.0, −1.0)
**Biomarkers**												
Serum creatine kinase, U/L												
n	15	15	11	9	6	6	5	5	5	5	4	2
% CFBL mean (SD)	−20.5 (35.38)	−29.9 (21.67)	−30.0 (35.59)	−35.3 (27.11)	−50.6 (20.18)	−46.5 (19.32)	−50.7 (14.70)	−35.9 (36.12)	−20.8 (8.84)	−25.3 (16.83)	−27.1 (32.43)	−23.7 (23.90)
CFBL median (Q1, Q3)	−22.9 (−44.0, −4.3)	−37.2 (−48.0, −14.1)	−47.7 (−55.1, −3.1)	−39.7 (−51.7, −35.5)	−48.6 (−61.0, −34.9)	−44.8 (−56.8, −35.1)	−53.7 (−59.1, −45.7)	−58.8 (−59.9, −17.0)	−23.0 (−23.1, −17.9)	−29.2 (−40.0, −7.8)	−31.0 (−54.5, 0.4)	−23.7 (−40.6, −6.8)
Urinary Hex4, mmol/mol creatine												
n	16	14	12	9	6	6	5	5	5	5	4	2
% CFBL mean (SD)	−36.11 (20.40)	−11.8 (68.57)	−35.1 (42.20)	−4.0 (82.57)	−40.7 (40.60)	−49.5 (48.06)	−39.3 (59.19)	−39.9 (44.68)	−15.6 (16.67)	−34.1 (14.08)	−36.5 (23.13)	−9.4 (1.71)
CFBL median (Q1, Q3)	−39.8 (−54.7, −15.3)	−33.7 (−57.8, −5.3)	−45.5 (−63.6, −16.4)	−29.1 (−46.4, 7.8)	−45.7 (−68.1, −21.9)	−63.3 (−75.9, −54.1)	−56.2 (−78.0, −33.3)	−43.8 (−73.4, −17.7)	−20.4 (−26.0, −2.2)	−34.8 (−40.6, −31.9)	−42.7 (−52.4, −20.5)	−9.4 (−10.6, −8.2)

6MWD 6-minute walking distance; *CFBL* change from baseline; *ERT* enzyme replacement therapy; *FVC* forced vital capacity; *Hex4* urine glucose tetrasaccharide; *MMT* manual muscle test; *SD* standard deviation

Supplementary Table S3 Summary of Subject and Physician Global Impression of Change (S/PGIC) at 48 months follow-up

	**Ambulatory ERT experienced**		**Non-ambulatory ERT experienced**	**Ambulatory ERT naïve**
	**Cohort 1 *N* = 7**	**Cohort 4 *N* = 6**	**Cohort 2 *N* = 5**	**Cohort 3 *N* = 6**
**SGIC; Overall physical wellbeing**				
Improved, *n* (%)	6 (85.7)	4 (66.7)	3 (60.0)	5 (83.3)
No change, *n* (%)	1 (14.3)	1 (16.7)	2 (40.0)	1 (16.7)
Declined, *n* (%)	0 (0)	1 (16.7)	0 (0)	0 (0)
**PGIC**				
Improved, *n* (%)	4 (57.1)	2 (33.3)	3 (60.0)	6 (100.0)
No change, *n* (%)	3 (42.9)	4 (66.7)	2 (40.0)	0 (0)
Declined, *n* (%)	0 (0)	0 (0)	0 (0)	0 (0)

ERT enzyme replacement therapy

Supplementary Table S4 Summary of pharmacokinetic outcomes for plasma signature peptide T09

Treatment group	N	C_{max}, µg/mL	t_{max}, h	$AUC_{0–t}$, µg·h/mL	$AUC_{tmax–24\ h}$, µg·h/mL	$AUC_{0–\infty}$, µg·h/mL	$t_{1/2\alpha}$, h	$t_{1/2\beta}$, h	CL_T, L/h
	**ERT-experienced patients (cohort 1)**								
Cipaglucosidase alfa 5 mg/kg	10	58.4 (19.1)	4.00 (3.00–4.10)	208 (18.1)	107 (27.1)	209 (18.0)	1.1 (11.3)	1.9 (19.3)	2.17 (17.0)
Cipaglucosidase alfa 10 mg/kg	11	135 (18.3)	4.00 (3.50–4.00)	533 (23.7)	288 (25.3)	537 (23.9)	1.3 (9.10)	1.6 (42.1)	1.66 (22.4)
Cipaglucosidase alfa 20 mg/kg	11	325 (13.5)	4.00 (3.50–4.00)	1,405 (16.2)	837 (19.4)	1,410 (15.9)	1.52 (9.2)	2.3 (38.7)	1.27 (17.8)
Cipaglucosidase alfa 20 mg/kg + miglustat 130 mg first dose	11	329 (14.3)	4.00 (3.40–4.10)	1,633 (17.2)	1,069 (19.3)	1,640 (16.9)	1.9 (10.6)	2.3 (17.3)	1.09 (18.8)
Cipaglucosidase alfa 20 mg/kg + miglustat 130 mg third dose	11	335 (15.4)	4.00 (3.40–4.30)	1,666 (19.1)	1,080 (20.9)	1,677 (18.6)	1.9 (22.0)	2.3 (21.1)	1.07 (18.6)
Cipaglucosidase alfa 20 mg/kg + miglustat 260 mg first dose	10	339 (12.9)	4.00 (3.50–4.00)	1,778 (17.6)	1,202 (18.5)	1,788 (17.2)	2.2 (19.1)	2.5 (21.6)	0.994 (21.8)
Cipaglucosidase alfa 20 mg/kg + miglustat 260 mg third dose	11	345 (18.5)	3.90 (3.40–4.00)	1,801 (19.9)	1,203 (23.4)	1,812 (20.8)	2.1 (15.9)	2.6 (19.2)	0.991 (22.4)
	**ERT-naïve patients (cohort 3)**								
Cipaglucosidase alfa 20 mg/kg + miglustat 260 mg first dose	6	342 (16.4)	4.00 (4.00–4.00)	1,854 (18.2)	1,235 (21.7)	1,857 (18.3)	2.2 (14.5)	2.5 (4.9)	0.740 (25.0)
Cipaglucosidase alfa 20 mg/kg + miglustat 260 mg third dose	6	323 (12.8)	4.10 (4.00–4.50)	1,772 (17.3)	1,153 (18.7)	1,774 (17.4)	2.2 (9.60)	2.5 (3.5)	0.768 (25.4)

C_{max} and AUC are reported as geometric mean (%CV), t_{max} is reported as median (range), and $t_{1/2}$ and CL_T are arithmetic means (%CV). *$AUC_{0–t}$* area under the plasma drug concentration–time curve from time zero to observed time (t); *$AUC_{0–\infty}$* area under the plasma drug concentration–time curve from time zero extrapolated to infinite time; *$AUC_{tmax–24\ h}$* area under the plasma–time curve from the time to reach maximum observed plasma concentration to 24 hours after the start of infusion; *CL_T* total clearance following intravenous administration; *C_{max}* maximum observed plasma concentration; *%CV* percentage coefficient of variation; *$t_{1/2\alpha/\beta}$* alpha/beta phase elimination half-life; *t_{max}* time to reach the maximum observed concentration

Supplementary Table S5 Summary of pharmacokinetic outcomes for plasma miglustat

Treatment group	N	C_{max}, ng/mL	t_{max}, h	$AUC_{0–t}$, ng·h/mL	$AUC_{0–\infty}$, ng·h/mL	$t_{1/2\beta}$, h	CL/F, L/h	V_Z/F, L
	**Cohort 1**							
Miglustat 130 mg First dose	11	1,527 (26.0)	3.47 (1.52–5.00)	11,759 (29.9)	12,611 (24.6)	6.13 (18.9)	10.5 (21.5)	93.4 (31.1)
Miglustat 130 mg Third dose	11	1,505 (23.9)	3.00 (1.50–4.00)	11,946 (24.6)	12,880 (25.7)	6.41 (28.6)	10.3 (21.7)	96.3 (38.6)
Miglustat 260 mg First dose	10	2,665 (31.8)	3.99 (1.98–5.00)	22,860 (33.4)	24,695 (33.8)	6.51 (16.2)	10.9 (27.5)	103.3 (31.9)
Miglustat 260 mg Third dose	11	3,089 (28.8)	3.00 (0.92–4.05)	23,492 (30.0)	24,938 (30.6)	5.97 (18.1)	10.8 (18.1)	93.9 (35.2)
	**Cohort 3**							
Miglustat 260 mg First dose	6	3,632 (23.0)	2.01 (0.95–3.00)	25,933 (11.6)	27,203 (11.0)	5.61 (21.0)	9.61 (11.7)	78.1 (24.2)
Miglustat 260 mg Third dose	6	3,000 (17.5)	2.60 (2.00–3.00)	24,413 (18.8)	25,735 (16.5)	5.77 (17.8)	10.2 (15.1)	86.3 (28.4)
	**Combined (cohort 1 and cohort 3)**							
Miglustat 260 mg First dose	16	2,993 (30.6)	3.00 (0.95–5.00)	23,967 (26.4)	25,607 (26.6)	6.17 (18.6)	10.4 (24.0)	93.8 (32.5)
Miglustat 260 mg Third dose	17	3,057 (29.1)	3.00 (0.92–4.05)	23,813 (25.9)	25,217 (25.8)	5.91 (17.5)	10.6 (22.4)	91.2 (32.6)

C_{max} and AUC are reported as geometric mean (%CV), t_{max} is reported as median (range), and $t_{1/2}$, CL/F and V_Z/F are arithmetic means (%CV). *$AUC_{0–t}$* area under the plasma drug concentration–time curve from time zero to observed time (t); *$AUC_{0–\infty}$* area under the plasma drug concentration–time curve from time zero extrapolated to infinite time; *CL/F* apparent total clearance of the drug from plasma after oral administration; *C_{max}* maximum observed plasma concentration; *%CV* percentage coefficient of variation; *$t_{1/2\beta}$* terminal elimination half-life; *t_{max}* time to reach the maximum observed concentration; *V_z/F* terminal phase volume of distribution following oral administration

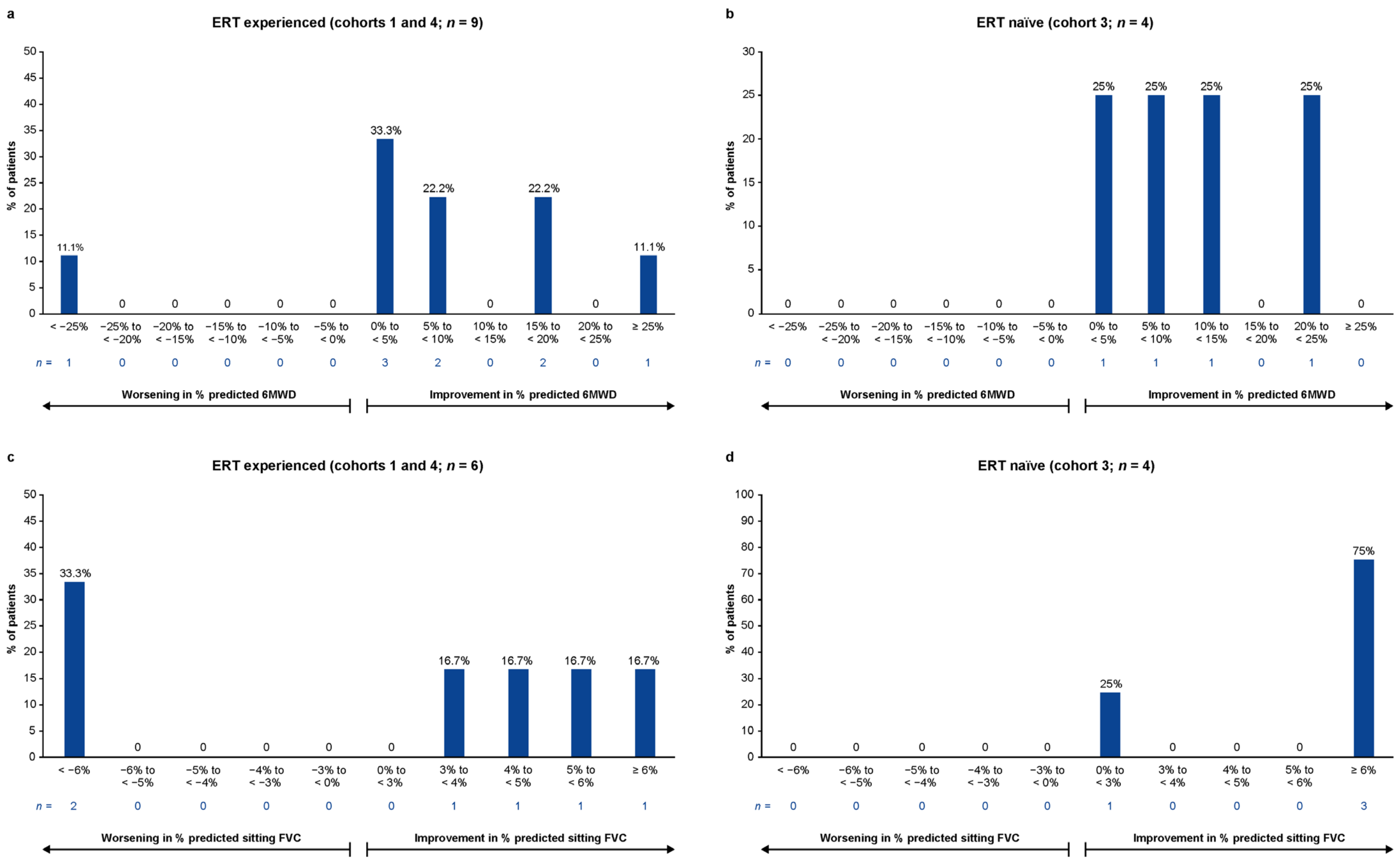

Supplementary Fig. S1 Proportion of patients with CFBL in % predicted 6MWD for ambulatory (a) ERT-experienced and (b) ERT-naïve patients and CFBL in % predicted FVC for ambulatory (c) ERT-experienced and (d) ERT-naïve patients at month 48.

Panels A and C are pooled data from cohorts 1 and 4 for ERT-experienced patients. Data were available for (a) *n* = 9, (b) *n* = 4, (c) *n* = 6 and (d) *n* = 4 patients. *6MWD* 6-minute walk distance; *CFBL* change from baseline; *ERT* enzyme replacement therapy; *FVC* forced vital capacity

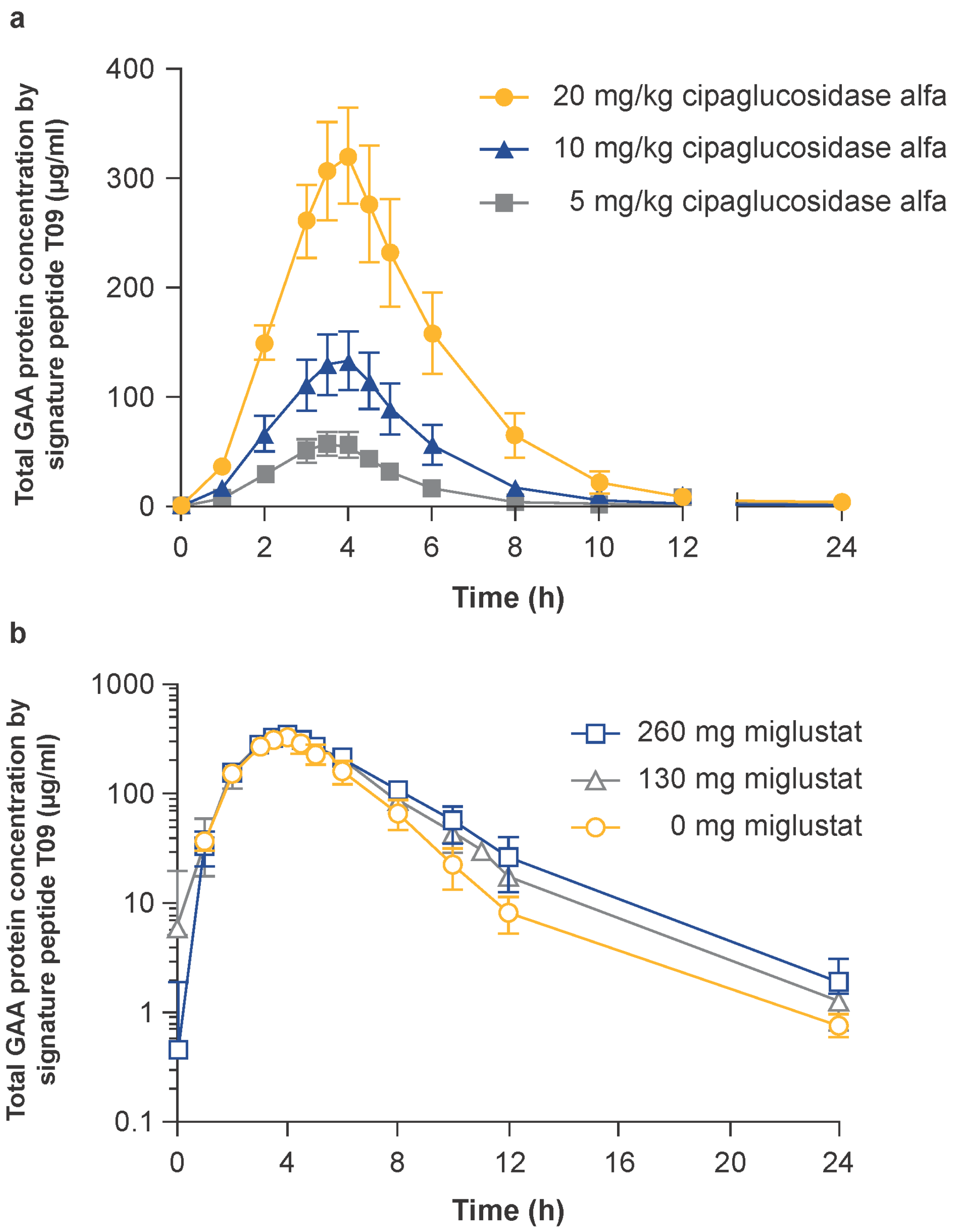

Supplementary Fig. S2 Plasma total GAA protein by signature peptide T09 concentration time profiles in ambulatory, ERT-experienced patients receiving cipaglucosidase alfa with and without miglustat.

Patients received a single ascending dose of cipaglucosidase alfa without miglustat (a) or 20 mg/kg cipaglucosidase alfa with and without ascending doses of miglustat (b). *AUC* area under the plasma drug concentration–time curve; *ERT* enzyme replacement therapy; *GAA* acid α-glucosidase; *h* hour

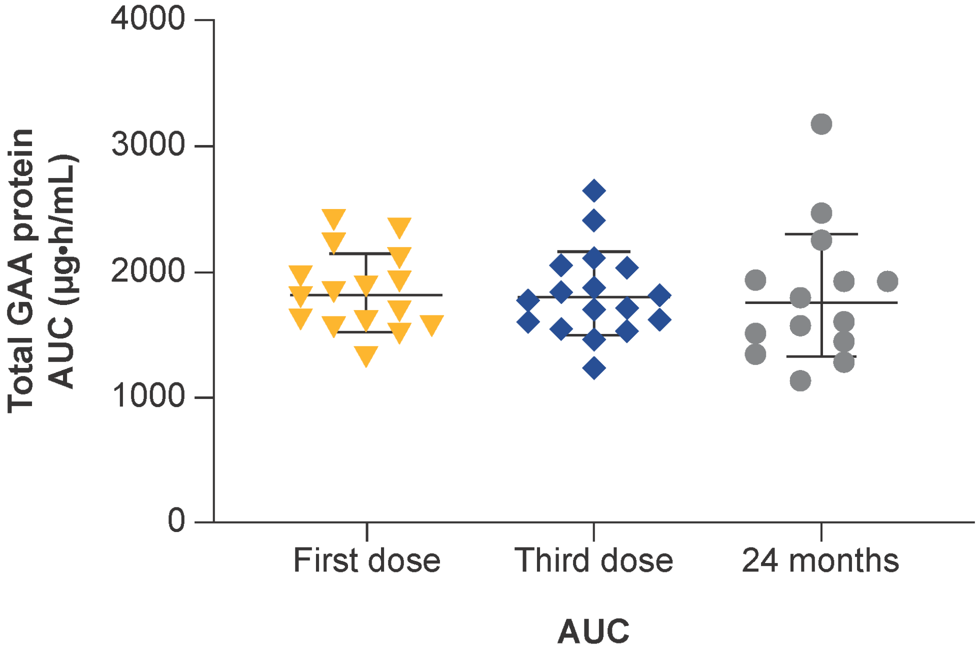

Supplementary Fig. S3 Plasma total GAA protein by signature peptide T09 concentration AUCs estimated by population PK analysis at 24 months and estimated by non-compartmental analysis following the first and third doses of 20 mg/kg cipaglucosidase alfa + 260 mg miglustat in cohorts 1 and 3.

AUC area under the plasma drug concentration–time curve; *GAA* acid α-glucosidase; *PK* pharmacokinetics
