# Supplementary material for: Long-term safety and efficacy of cipaglucosidase alfa plus miglustat in individuals living with Pompe disease: an open-label phase I/II study (ATB200-02)
Source: J Neurol. 2023 Dec 6;271(4):1787–801. doi: 10.1007/s00415-023-12096-0 (PMC10973052; doi:10.1007/s00415-023-12096-0)
Supplement: Supplementary file 2 — Supplementary file2 (PDF 1954 KB) [file 415_2023_12096_MOESM2_ESM.pdf]

Long-term safety and efficacy of cipaglucosidase alfa plus miglustat in individuals living with Pompe disease: an open-label Phase I/II study (ATB200-02)

Barry J. Byrne et al

What is Pompe disease?

Pompe disease is a rare, inherited, multisystemic disorder that leads to **muscle weakness** and **breathing difficulties** over time. It is caused by **the lack of an enzyme called acid α-glucosidase (GAA)**, typically found inside muscle cells.

In healthy muscle cells, the **GAA enzyme breaks down the sugar glycogen into glucose.**

But, in Pompe disease, the deficiency in GAA enzyme activity means that **glycogen cannot be broken down** and **it builds up inside muscle cells**, which **causes damage** leading to muscle weakness and breathing difficulties over time.

Why did we do this study?

Treatment considerations

Current therapies aim to replace the missing GAA enzyme in the muscle cells of people living with Pompe disease. These are called enzyme replacement therapies (ERTs) and are associated with the following key challenges:

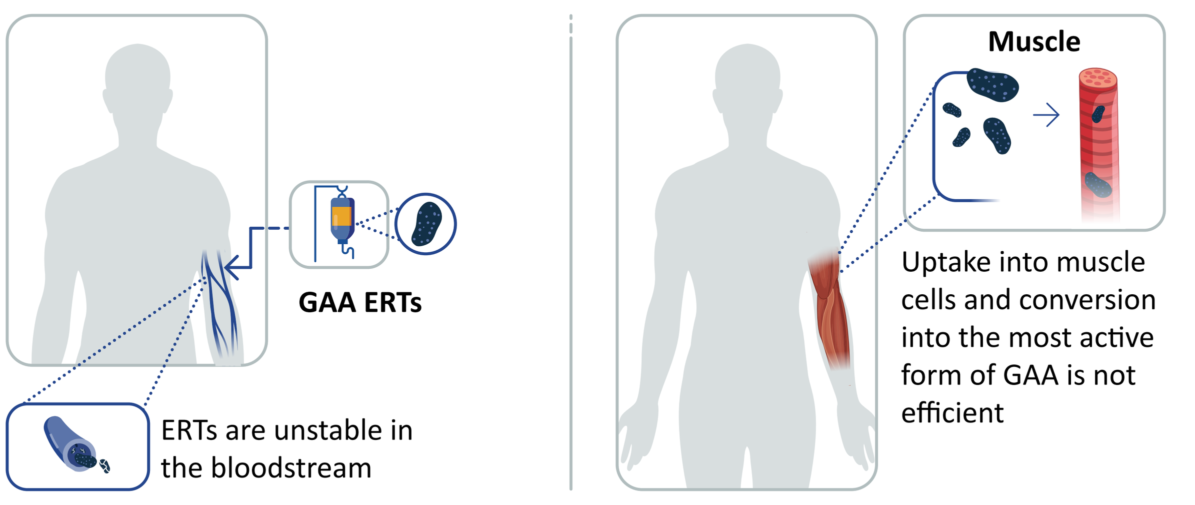

Novel therapy: cipaglucosidase alfa + miglustat

Cipaglucosidase alfa + miglustat is a novel two-component therapy designed to minimize breakdown in the bloodstream before it reaches the target muscle cells and to improve uptake into muscle cells

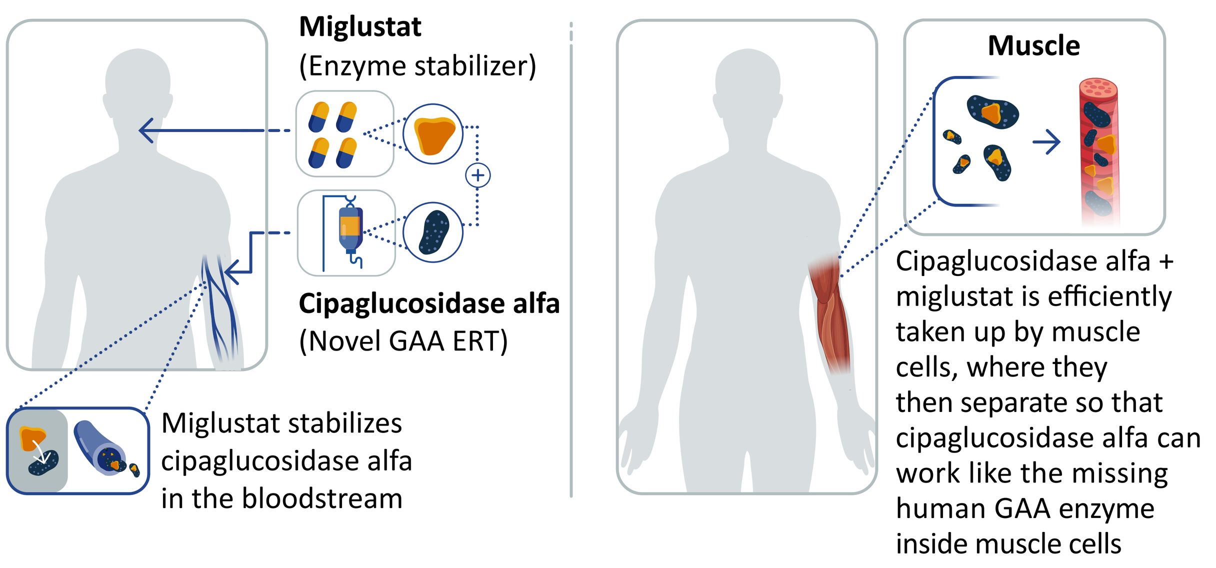

We are doing this study to investigate whether cipaglucosidase alfa + miglustat improves the measurements of disease progression in people living with Pompe disease

How did we do this study?

We enrolled three groups of ambulatory participants

Group 1	Group 4	Group 3
2–6 years	≥ 7 years	ERT naïve
ERT experienced	ERT experienced	Median age = 51.0 years
Median age = 50.0 years	Median age = 43.0 years	
n = 9 (male), *n* = 2 (female)	*n* = 4 (male), *n* = 4 (female)	*n* = 1 (male), *n* = 5 (female)

ERT-experienced group (pooled group)
Total enrolled = 17
Ages = 18–75 years

ERT-naïve group
Total enrolled = 6
Ages = 18–65 years

Participants were enrolled at different times; therefore, not everyone has reached the later time points of this ongoing study

All participants received cipalglucosidase alfa + miglustat every 2 weeks

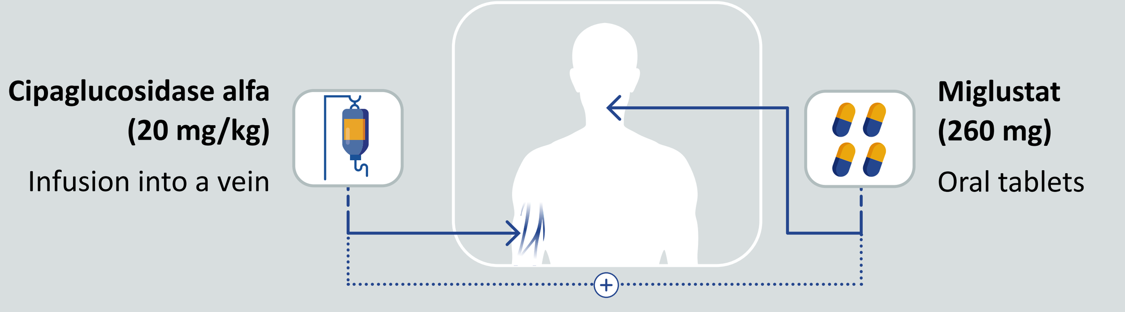

We checked standard Pompe disease assessments in participants at regular time intervals

- We measured the **motor function, lung function, urine marker levels for glycogen build-up (hexose tetrasaccharide [Hex4]), blood marker levels for muscle damage (creatine kinase [CK])**, and **medical issues** of enrolled participants at different time points over the course of the trial
- The first measurements were taken before treatment began (baseline) and other measurements were taken at different time points up to 48 months after treatment with cipaglucosidase alfa + miglustat
- This allowed us to investigate how cipaglucosidase alfa + miglustat treatment impacts the assessment outcomes over time

Months: Baseline, 6, 12, 24, 36, 48, Ongoing	**% predicted[a] 6-minute walk distance (6MWD)**	**% predicted[a] forced vital capacity (FVC)**	**Markers of muscle damage (CK) and glycogen levels (Hex4)**	**Safety profile**
	To assess **motor function**, we measured how far participants could walk in 6 minutes	To assess **lung function**, we used a spirometer to measure participants' FVC, which is a measure of how much air can be expelled from the lungs after taking a deep breath	**CK** and **Hex4** are markers for **muscle damage** (CK)[1] and **glycogen build-up** (Hex4).[2] Higher blood levels of CK and higher urine levels of Hex4 typically indicate more muscle damage and higher glycogen build-up[1, 2]	The **safety** of the study treatment was evaluated on an ongoing basis

[a]6MWD and FVC are calculated as percent (%) predicted, which standardizes the results based on gender, age, height and weight for 6MWD, and gender, age, height and race for FVC

What have we found so far?

6MWD: a measure of motor function

Participants treated with cipaglucosidase alfa + miglustat **walked further in 6 minutes** compared with before starting treatment, and this improvement was **maintained for up to 48 months**

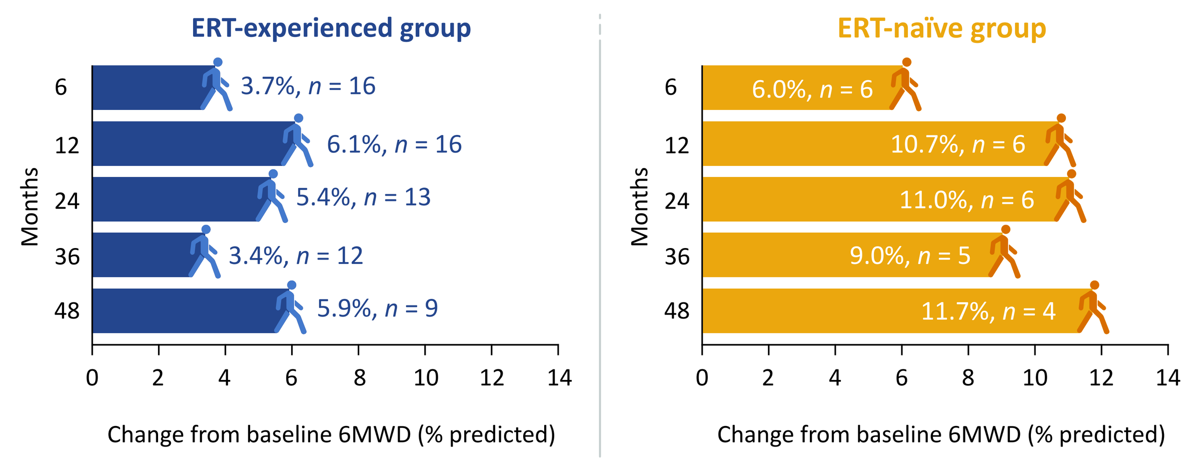

FVC: a measure of lung function

The **lung function** of participants treated with cipaglucosidase alfa + miglustat **stabilized or improved** compared with the baseline, and this was **maintained for up to 48 months**

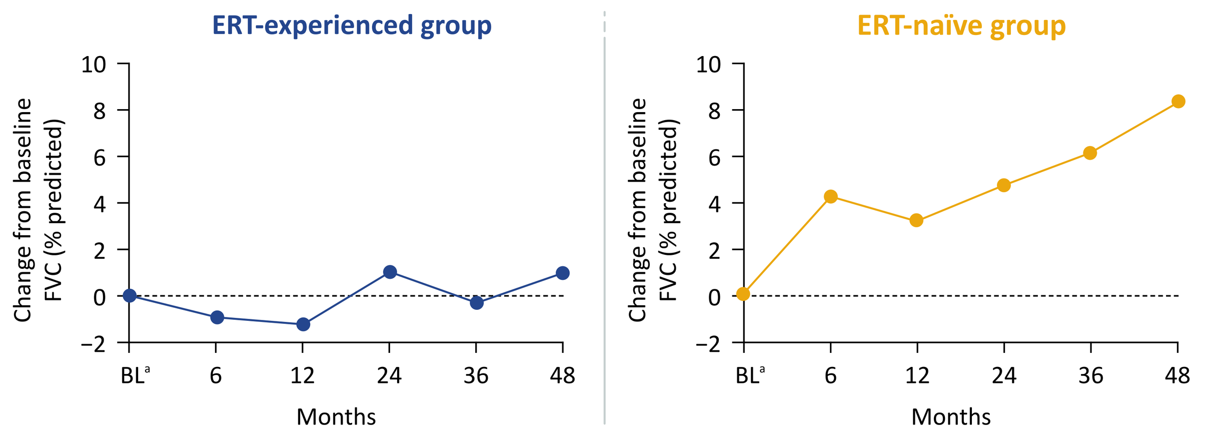

[a]*BL* baseline

Blood levels of CK: a marker for muscle damage

Participants treated with cipaglucosidase alfa + miglustat had **lower levels of CK**, a blood marker for muscle damage, which may indicate **less damage to muscles**

CK levels in ERT-experienced group
CK mean % change from baseline levels ranged from −4.5 to −38.5 over the study period so far

Lower levels of CK may indicate decreased muscle damage

CK levels in ERT-naïve group
CK mean % change from baseline levels ranged from −35.9 to −57.9 over the study period so far

Urine levels of Hex4: a marker for glycogen build-up

Participants treated with cipaglucosidase alfa + miglustat had **lower levels of Hex4**, a urine marker for glycogen levels, indicating that the treatment is **breaking down glycogen in the muscle cells**

Hex4 levels in ERT-experienced group
Hex4 mean % change from baseline levels ranged from −4.0 to −41.2 over the study period so far

Lower levels of Hex4 may indicate reduced glycogen build-up

Hex4 levels in ERT-naïve group
Hex4 mean % change from baseline levels ranged from −28.6 to −49.5 over the study period so far

Safety profile

Most medical issues experienced by participants treated with cipaglucosidase alfa + miglustat were **mild or moderate** in severity and did not lead to study withdrawal

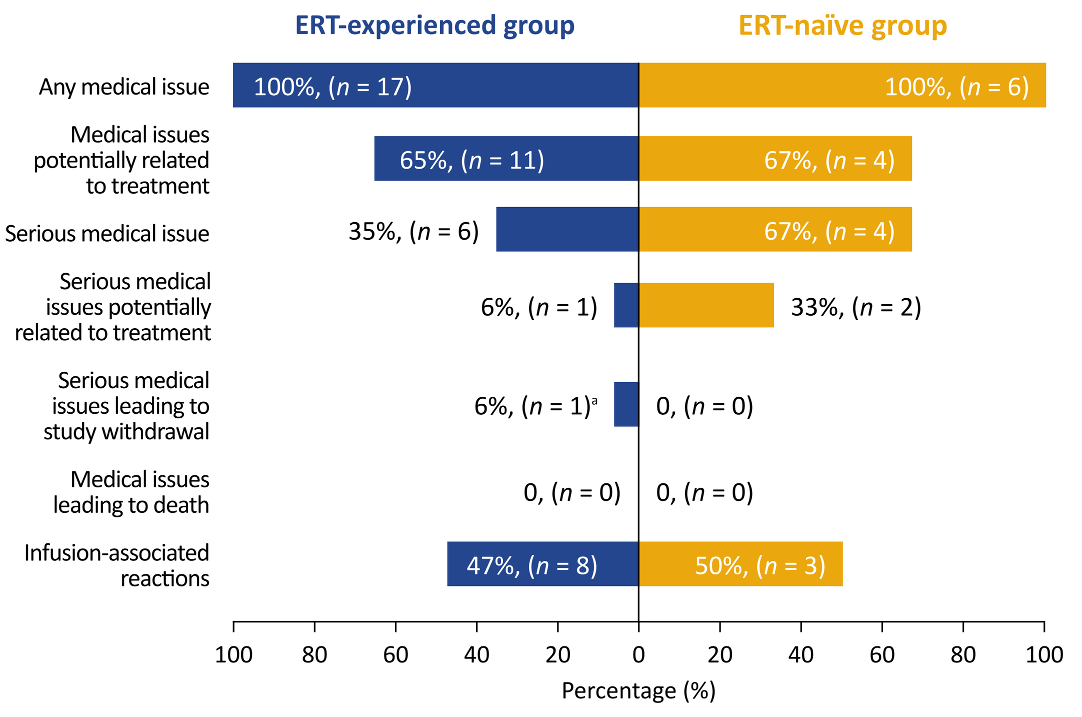

[a]One case of diffuse large B-cell lymphoma not related to treatment

The most common **medical issues** that occurred after participants started the study treatment were:

- **Fall** 70%, (*n* = 16)
- **Common cold** 57%, (*n* = 13)
- **Headache** 52%, (*n* = 12)
- **Muscle pain** 52%, (*n* = 12)
- **Diarrhea** 44%, (*n* = 10)

What do our results mean for people with Pompe disease?

Participants treated with cipaglucosidase alfa + miglustat had overall improved outcomes compared with before treatment

Participants **could walk further in 6 minutes** compared with before treatment. This indicates that the **motor function** of participants **improved** over the course of the trial so far

The **lung function** of participants **improved or stabilized** compared with before treatment. This indicates that, on average, participants' **breathing improved or did not get worse** during the trial so far

A blood **marker for muscle damage (CK)** and a urine **marker for glycogen build-up (Hex4)** both decreased in participants compared with before treatment started, which could indicate **an improvement in disease progression**

Most **side effects** were **mild or moderate in severity** and did not lead to withdrawal from the study. This indicates that cipaglucosidase alfa + miglustat is **well tolerated overall** by participants enrolled in this trial and has a safety profile consistent with currently available ERTs

This summary accompanies the manuscript titled 'Long-term safety and efficacy of cipaglucosidase alfa plus miglustat in individuals living with Pompe disease: an open-label Phase I/II study (ATB200-02)'.
[1]Brancaccio P et al (2010) Clin Chem Lab Med 48:757–767; [2]An Y et al (2005) Mol Genet Metab 85:247–254.
Clinical trial identifier: NCT02675465.
This study was supported by Amicus Therapeutics, Inc., Philadelphia, PA, USA.
Editorial assistance was provided by Sophie Austin, PhD, at Cence (an AMICULUM® agency), funded by Amicus Therapeutics, Inc.
